# Ecological consequences of colony structure in dynamic ant nest networks

**DOI:** 10.1002/ece3.2749

**Published:** 2017-01-24

**Authors:** Samuel Ellis, Daniel W. Franks, Elva J. H. Robinson

**Affiliations:** ^1^ Centre for Research in Animal BehaviourUniversity of ExeterExeterUK; ^2^ York Centre for Complex Systems Analysis & Department of BiologyUniversity of YorkYorkUK

**Keywords:** dynamic networks, polydomy, red wood ants, social group, survival analysis

## Abstract

Access to resources depends on an individual's position within the environment. This is particularly important to animals that invest heavily in nest construction, such as social insects. Many ant species have a polydomous nesting strategy: a single colony inhabits several spatially separated nests, often exchanging resources between the nests. Different nests in a polydomous colony potentially have differential access to resources, but the ecological consequences of this are unclear. In this study, we investigate how nest survival and budding in polydomous wood ant (*Formica lugubris*) colonies are affected by being part of a multi‐nest system. Using field data and novel analytical approaches combining survival models with dynamic network analysis, we show that the survival and budding of nests within a polydomous colony are affected by their position in the nest network structure. Specifically, we find that the flow of resources through a nest, which is based on its position within the wider nest network, determines a nest's likelihood of surviving and of founding new nests. Our results highlight how apparently disparate entities in a biological system can be integrated into a functional ecological unit. We also demonstrate how position within a dynamic network structure can have important ecological consequences.

## 1 Introduction

An individual's access to resources is strongly influenced by its position in the environment relative to that resource. This can have important behavioral consequences; for example, optimal foraging strategies have evolved to make best advantage of available resources, given an individual's position in the environment (Ydenberg, 2007). This is particularly true in species such as social insects, which form nests that are spatially fixed (at least in the short term). The position of a nest in the environment is likely to affect access to resources and ultimately the fitness of the individuals within the nest (McGlynn, 2012).

Many ant species inhabit multiple spatially separated, but socially connected nests, a strategy called polydomy (Debout, Schatz, Elias, & Mckey, 2007; Robinson, 2014). Nests within a polydomous system often exchange resources (e.g., Buczkowski, 2012; Ellis, Procter, Buckham‐Bonnett, & Robinson, in press; Hoffmann, 2014). A nest's access to resources will depend not only on its location within the foraging environment but also on its position relative to other nests. For example, in polydomous wood ant (*Formica lugubris*) colonies food and other resources are transported through the colony by workers traveling along trails between nests (Ellis, Franks, & Robinson, 2014; Ellis & Robinson, 2015, 2016). The combined nests and trails of a polydomous wood ant colony therefore act as a resource redistribution network: food resources are transferred along the trails between pairs of nests, resulting in colony‐level redistribution of resources organized at a local level (Ellis & Robinson, 2016; Ellis et al., 2014). Wood ants’ major source of food is honeydew, a spatially and temporally stable resource (Domisch, Risch, & Robinson, 2016). For a worker, therefore, access to food will depend not only on their nests’ location within the stable foraging environment but also on their nests’ position in the nest network structure. Workers from the same colony, but inhabiting different nests, therefore have different access to resources. However, the ecological consequences of this differential access to resources of nests within the network, and the effect that this differential access has on the structure of the colony, are unclear.

In a polydomous colony, there are several possible ecological consequences of a nest's access to resources. For example, a nest's survival, i.e., its continued inhabitation, is likely to depend on its ability to access enough resources to sustain the ants within the nest. Similarly, workers within a nest may be influenced by access to resources when founding new nests. In polydomous wood ant colonies, new nests are often established by budding: During budding, workers and queens leave a nest on foot to found a new nest (Bourke & Franks, 1995; Ellis & Robinson, 2015). It would be expected that the decision of ants within a nest to bud a new nest is influenced, positively or negatively, by their nest (the founder nest's) access to resources (Holway & Case, 2000; Lanan, Dornhaus, & Bronstein, 2011; Sorvari & Hakkarainen, 2005). It is important to note that both of these traits: survival and budding, are inherently time dependent and need to be studied in a dynamic framework.

The ecological interdependence of nests will define the nature of the polydomous system. In a monodomous colony (a colony inhabiting a single nest), the survival and budding of a nest are affected only by properties inherent to that nest, such as its size and location in the environment. Nests within a polydomous system may similarly survive and bud based only on their inherent properties, with no ecological consequences of the nest network structure. Survival and budding based only on inherent properties of a nest would suggest that there is a low level of integration between nests in the system and that a polydomous colony is simply a cluster of mutually nonaggressive nests and not part of a single cooperative and functional unit. In contrast, if the nests of a polydomous system are part of the same functional unit, the survival and budding of each nest will be affected not only by inherent nest properties, but also by either its position in the colony nest network or more general colony‐level effects.

In this study, we investigate how the survival and budding of nests in polydomous colonies are affected by three levels of organization: (i) attributes of the individual nest, (ii) position of the individual nest within the network, and (iii) properties common to the whole network. The ecological consequences of differential access to resources within a polydomous colony will give important insights into how polydomous colonies are structured and, more generally, the potential importance of an individual's position within a dynamic network.

## 2 Materials and Methods

### 2.1 Study species and study site

We investigated the dynamics of the nest networks of the polydomous red wood ant *Formica lugubris*, a member of the ecologically important *F. rufa* species group (Stockan & Robinson, 2016; Stockan et al., 2016). Wood ants are the dominant invertebrate predator in their environment; they hunt and scavenge for a variety of invertebrate prey, including other ant species (Domisch et al., 2016; Mabelis, 1984; Savolainen & Vepsäläinen, 1988). However, the majority of food for red wood ant colonies is provided by foraging for honeydew collected from sap‐feeding hemipterans in the canopy (Domisch et al., 2016; Rosengren & Sundström, 1991). These hemipteran colonies provide a spatially and temporally stable food source for the ants. Analysis of the structure of wood ant nest networks has highlighted the importance of honeydew transport in structuring the colony trail structure (Ellis et al., 2014). Detailed observation of the trails between wood ant nests has also suggested that honeydew is the main resource being transported along the internest trails (Ellis & Robinson, 2015, 2016). This study was conducted at the Longshaw Estate in central England. *Formica lugubris* is the only *F. rufa* group (the red wood ants) species at this site. The *F. rufa* group are the dominant ants in European woodlands (Johansson & Gibb, 2016; Savolainen & Vepsäläinen, 1988). Their only significant competitors are therefore other wood ant species (Johansson & Gibb, 2016). The absence of other wood ant species at this site means the *F. lugubris* have no significant interspecific competitors. The site is a mixture of woodland pasture and historic plantations. Wood ants construct nest mounds from pine needles and other material available in the leaf litter. In England, *Formica lugubris* are polygynous and each nest of the colony is likely to contain multiple queens (Ellis & Robinson, 2014; Procter et al., 2016). Scots pine (*Pinus sylvestris*), oak (*Quercus sp*.), larch (*Larix sp*.) sycamore (*Acer pseudoplatanus*), and silver birch (*Betula pendula*) are the most common tree species at the site. Wood ant colonies at the site show no preference for particular tree species, neither do they show any temporal variation in tree species preference (Samuel Ellis *unpublished*). The lack of preference for particular tree species suggests that the resources available from the different tree species at the site are approximately equal. Their large size, stable food sources, and lack of significant predators and competitors mean that wood ant nests are often present in the same location for a long period of time (Risch, Ellis, & Wiswell, 2016; Robinson & Robinson, 2008).

### 2.2 Network mapping

**Figure 1:**
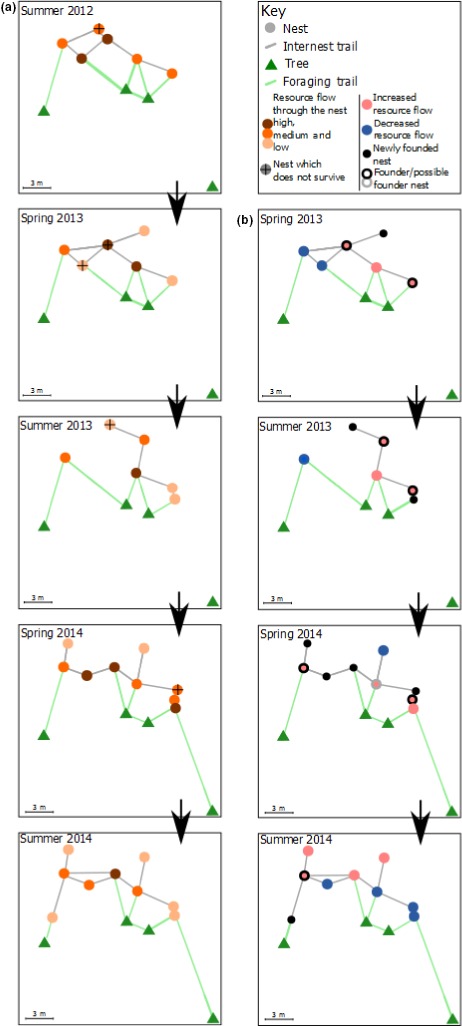
Timeseries of colony IIb used in this study to illustrate some of the ecological consequences of nest network position in polydomous *Formica lugubris* colonies. A small network was chosen for the purpose of simplicity. (a) Colony IIb at the five time points used in this study. Nests are represent as circles and categorized as having a low resource flow (normalized betweeness of less than 0.25: pale orange) a medium level of resource flow (normalized betweeness of more than 0.25 and less than 0.75: orange), and a high level of resource flow (normalized betweeness of greater than 0.75: deep orange/brown). Nests with a black cross are those which will not survive until the next time point (no data for after summer 2014, so no nests are marked as being abandoned). Green triangles represent trees. The lines between points represent foraging (green) and internest trails (gray). Nests with a low or medium flow were abandoned more often than those with a high flow. (b) Colony IIb at 4 time points in the study. As above, circles represent nests. Circle color represent the change in flow through the nest since the last time point; blue indicates a decrease or no change in flow of resources since the last time point. Pink shows nests which have increased in resource flow since the last time point. Black circles are the newly founded nests. Black outlines represent nests from which a new nest(s) has been founded (founders); gray outlines represent possible founders. Nests with an increased resource flow were more likely to found new nests than those with a static or decreased resource flow

We represented the polydomous colonies as networks, with the nests and trees as nodes and the internest and foraging trails as edges (e.g., Latty et al., 2011; Cook, Franks, & Robinson, 2014; Ellis et al., 2014; Figure 1). Wood ants form clear above‐ground trails between nests (internest trails) and between nests and trees (foraging trails). The trails consist of workers traveling along fixed paths often transporting resources, predominantly honeydew, invertebrate prey, and brood (Ellis & Robinson, 2015, 2016). Workers show very high fidelity to trails, rarely switching between trails once they have been recruited (Ellis & Robinson, 2016; Gordon, Rosengren, & Sundström, 1992). We define a polydomous colony as two or more nests connected by internest trails (Ellis et al., 2014). Our definition of a colony is, therefore, based on functional resource exchange between nests, rather than on the basis of aggression or relatedness.

We use the same mapping method employed by Ellis et al. (2014) previously at this site to map the same colonies over 4 additional time points over the next 2 years. For each colony, at each mapping time point, we recorded the spatial and topological layout of the nests, trees, and trails. For the trails, we measured the length of the trail, compass direction of the trail, and the traffic on the trail. The traffic on the trail was measured as the length of trail needed to find 10 workers, which can be converted into number of ants per cm of trail and then number of ants on the entire length of the trail. The advantage of basing trail traffic on distance needed to find ants, rather than a rate‐based measure, is that it is not reliant on the speed at which the ants are moving, which is strongly affected by the ambient temperature (Rosengren, 1977). Ant traffic is a measure of trail strength based only on the number of ants passing along the trail; however, this is likely to be affected by the number of workers available to travel along the trails. Trail weight is a measure of trail important to a particular nest, relative to the populations of the nests being connected. We calculated each trail's weight by dividing the total number of ants on the trail by the mean population of the nests connected by the trail (Ellis et al., 2014). For foraging trails, the weight of the trails are relative to the population of the foraging nest (Ellis et al., 2014).

For each mapping of each colony, we also estimated the populations of the nests (Chen & Robinson, 2013), measured the canopy cover over the nests, and recorded the species of the trees used for foraging. The worker population in a nest was estimated from the volume of the nest mound, calibrated at this site with a mark–release–recapture measure (Chen & Robinson, 2013; Ellis et al., 2014). Canopy cover was estimated on the basis of digital photographs taken from vertically above each nest (Ellis et al., 2014). Each map was used to construct a spatially embedded network of the colony, with edges weighted by trail strength, and the node properties of nest population (hereafter: nest size), distance to the nearest tree, and canopy cover. We examined how the nest networks of thirteen polydomous wood ant colonies changed over time. Thirteen of the largest colonies at the site were studied, chosen from a preliminary survey in May 2012 (Table 1; Appendix S1). The colonies were first fully mapped in late August 2012 (analyzed as static networks in Ellis et al., 2014). For the next 2 years (2013 and 2014), the colonies were fully mapped (using the methods outlined above) twice per year: once in late spring and again in late summer. Each remapping was performed blind, without reference to the maps of previous time points. Wood ants show seasonal activity patterns: They are quiescent over winter, beginning foraging activity (and producing sexual offspring) in late spring, and continuing foraging throughout the summer and early autumn (Maeder et al., 2016). Remapping colonies in late spring and again in late summer therefore represents the beginning and the height of the foraging season, respectively. The timing of the late spring mapping was dependent on the timing of spring in each year and was not performed until temperatures were high enough that both foraging and internest trail activity were being performed (Rosengren, 1977). Late summer mapping was always performed in the second half of August. Wood ant activity can be dependent on temperature and weather conditions so all colonies were mapped in warm, dry conditions.

**Table 1:** Details of the colonies used in this study. Numbers refer to the nests present in the colony at that timepoint. Spring refers to late May (the beginning of the foraging season) and summer in late August (the peak of the foraging season). Net change in nests describes the difference in number (and percentage) of nests in the colony between summer 2012 and summer 2014. Average nest population (nest size in the text) refers to the mean number of ants predicted to be in the nests of each colony

	Total number of nests					Net change in number of nests	Average nest population (range)
	2012‐Summer	2013‐Spring	2013‐ Summer	2014‐Spring	2014‐Summer		
I	21	16	15	11	14	−7 (−33.3%)	78,780 (625–1,791,617)
IIa	4	3	4	4	4	0 (0%)	22,941 (290–93,883)
IIb	6	6	6	9	9	+3 (+50%)	17,601 (1560–62,641)
III	12	12	8	9	16	+4 (+33.3%)	35435 (156–166,815)
IV	12	9	6	7	7	−5 (−41.6%)	36,588 (851–185,500)
V	14	11	10	8	2	−12 (−88%)	29,272 (522–265,005)
VI	14	12	12	13	11	−3 (−21%)	18,827 (119–110,039)
VII	7	7	4	5	8	+1 (+14%)	21,109 (285–114,238)
VIII	6	3	4	6	4	−2 (−33.3%)	54,255 (210–384,166)
IX	9	11	17	11	15	+6 (+66.6%)	32,681 (68–288,380)
X	13	8	10	9	8	−5 (−38%)	18,423 (160–90,064)
XI	20	15	10	10	17	−3 (−15%)	10,175 (89–130,860)
XII	6	6	3	8	3	−3 (−50%)	74,528 (1805–240,329)

### 2.3 Analysis

We are interested in how dynamic properties of nests within polydomous colonies are influenced by their inherent attributes (nest attributes), their position within the nest network (network position), and attributes shared with the whole colony (colony attributes; summarized in Table 2). All network analysis was performed in R (R Development Core Team 2011) using the “igraph” package (Csardi & Nepusz, 2006).

**Table 2:** The nest attribute, network position, and colony attribute variables used to investigate the ecological consequences of a nest position in a polydomous colony

Nest attributes	Network position	Colony attributes
The *population of the nest*. Calculated based on nest volume.	*Nest betweenness*. Calculated from the weighted network maps, including both trees and nests.	*Worker:forager ratio*. The total population of the colony (the sum of all nest populations) divided by the instantaneous number of workers on foraging trails (from the trail strengths).
The *canopy cover* above the nests. Collected using digital photographs of the canopy above the nest.		
The *distance to the nearest tree*. Calculated as the linear distance (i.e., not along foraging trails) from a nest to the nearest tree.		

Nest attributes are those based directly on inherent attributes of the nest. The population of a nest (hereafter nest size), canopy cover over the nest, and the distance from the nest to the nearest tree all have been shown to be ecologically important for wood ants (Chen & Robinson, 2014) and therefore have the potential to influence the survival and reproduction of nests.

Network position properties depend on a nest's location in the colony nest network. Resource exchange between the nests of a polydomous wood ant colony is based on workers from a given nest traveling along internest trails to neighboring nests, collecting honeydew, and then returning to their original, home, nest (Ellis & Robinson, 2016). This mechanism is based on local resource exchange, between neighboring nests, without reference to the efficiency of colony‐level resource redistribution (Ellis et al., 2014). A resource exchange mechanism based on workers from a given nest treating other nests as food sources has the potential to result in resource exchange through the entire colony (Cook, Franks, & Robinson, 2013; Schmolke, 2009). In a system based on local resource exchange, the quantity of resource available to a given nest can be represented as the flow through that nest: In wood ant colonies, resources flow from the trees, and then in some cases through the internest trail network, to the nests. Flow through a node in a network can be measured as betweeness centrality. Betweeness is a measure of the total number of shortest paths between pairs of nodes in the network which pass through a particular node (e.g., Croft, James, & Krause, 2008; Whitehead, 2008). We correct for igraph's reverse treatment of weighted values by inverting the strength of trails for centrality analysis. Our mapped networks include both the nests of the colony and the trees on which they are foraging, i.e., the analyzed networks contain both the colony and its foraging environment. In our polydomous networks, all trees are at the end of a network on their own branch and therefore have a betweeness of zero. In contrast, the betweeness of a nest is based on the number of shortest paths passing through it, including those from trees to other nests in the network. Betweeness can therefore act as a measure of (potential) resource flow through a particular nest, dependent on that nest's pattern of trails connected to other nests and trees in the network. We used a weighted measure of betweeness to account for the number of ants on a trail, given the size of the connected nests (trail weight). To allow comparison between networks for each colony, the betweeness was normalized to the largest value within each network (e.g., Lusseau & Newman, 2004).

Colony attributes are those which are shared by all the nests within a nest network. At the colony level, we are interested in how the amount of resources collected by the entire colony influences the survival and budding of the nests. We use the number of ants on foraging trails as a measure of a colony's foraging effort. The number of ants on foraging trails can be calculated by multiplying the ants per cm for every foraging trail by the length of that foraging trail and then summing these values for the whole colony. This foraging metric is a measure of the resource acquisition effort of the entire colony, not a count of the number of foragers in the colony. The ratio of the total population of the colony (summed size of all the nests in the colony) to the foraging effort of the colony, hereafter worker:foraging ratio, gives an estimate of foraging effort per worker in the colony. A low worker:foraging ratio suggests a high foraging effort per worker, whereas a high worker:foraging ratio indicates a low foraging effort per worker. We use the worker:foraging ratio as a measure of colony‐level resource acquisition. We investigate how worker:foraging ratio predicts various survival, population change, and budding of nests (see below) to see how this colony‐level measure of resource acquisition compares to the network position‐based resource acquisition measure: normalized betweeness.

Internest trails can also have inherent, within‐network, and colony attributes. An important inherent trait of an internest trail is the ant traffic on that trail. Ant traffic along a trail does not take into account the size of the nests connected by the trails. Trail weight takes into account the size of the nests being connected by the trails. The persistence of an internest trail is likely to be affected by the properties and network position of the nests which they join. Similarly, the number of internest trails and foraging trails associated with the two nests that an internest trail connects can also be considered a within‐network attribute. The betweeness of a trail, unlike the betweeness of a nest, is a colony‐level effect. Trail betweeness is a colony attribute because it represents the importance of a trail to colony‐level resource flow, rather than the amount of resources passing through a particular nest.

An underlying assumption of our measures of both network position properties and colony‐level properties is that the level of traffic on a foraging or internest trail is representative of the quantity of resources (specifically honeydew) being transported along this trail. This assumption is based on data showing that (i) the strength of an internest trail is positively related to the level of foraging being performed at each end of the trail (Ellis et al., 2014), (ii) 70% of internest journeys involve transport of honeydew (Ellis & Robinson, 2016), and (iii) the presence of internest trails predicts transport of resources between nests (Procter et al., 2016).

### 2.4 Nest survival

We are interested in which factors (nest attributes, network position, or colony attributes) influence the survival of a nest in a polydomous wood ant colony. We use survival analysis, adapted for use with network data, to investigate the factors influencing nest survival. Survival analysis is used to describe the time until an event occurs; for our purposes, the event in question is that a nest is abandoned (Kleinbaum & Klein, 2012). Nest abandonment can be inferred from the colony maps by the absence of a previously present nest at the next time point. The advantage of using survival analysis rather than more conventional statistical techniques is that censored data can be included. Censored data occur when some information is known about an individual, for example when a nest is founded, but not other information, for example when it is abandoned. This is useful for our data as many nests survive longer than our study period. Survival analysis allows us to investigate how the survival of a nest changes with time in relation to network dynamics.

We used an extended Cox proportional hazard (Cox PH) model to investigate the effect of explanatory variables (X_n_) on the hazard potential, *h(t)* (Equation (1) for the basic Cox PH model). The hazard potential is the instantaneous potential per unit time that a nest (or trail) is abandoned, given that the nest (or trail) has survived up to time *t* (Kleinbaum & Klein, 2012). The survival function, *S(t)*, describes the probability that a nest survives longer than a given time *t*. The extended Cox PH model allows time‐dependent explanatory variables to be included in the analysis (Kleinbaum & Klein, 2012).

(1) $$h\left(t,\;X\right)=h_{0}\left(t\right)e^{\sum_{i=1}^{p}\beta_{i}X_{i}}$$

$$X=(X_{1},\;X_{2},\ldots X_{p})$$

In the extended Cox PH models reported in this study, the explanatory variables (*X*_*p*_) were the nest attribute, network position, or colony attribute variable(s) being investigated. Colony was also used as an explanatory variable in all models. When the model was used to describe the survival of a trail, the survival of the nests associated with the trail was used as an additional explanatory variable. The presence of the nests which bound the trail is, clearly, vital to the survival presence of the trail itself and was always highly significant.

Survival models assume independence of data, an assumption that is violated by network data. Therefore, we constructed a null model based on the quadratic assignment procedure using 10,000 node attribute permutations (Croft, Madden, Franks, & James, 2011). We then measured the experimental test statistic against this null distribution to derive statistical significance. Permutations were constrained within each map (i.e., within each colony map from a particular time point). All reported statistics associated with survival were based on the quadratic assignment procedure. For some analyses, the smallest colonies (IIa and VIII) were not included because the lack of variation prevented the model defining the confidence intervals; this is indicated in the text by lowered values of *n*. Survival analysis was performed in R using the “survival” package (Therneau, 2012).

### 2.5 Nest budding

New nests were often founded within the polydomous colonies used in this study. A nest that was not present at the previous time point was considered to be newly founded. We used our colony layout maps (described above) to infer which nest acted as the founder of the newly founded nest. We refer to a nest from which a new nest is budded as its natal nest. To infer which nests are the natal nests, we assumed that (i) newly budded nests stay connected to their natal nest by a trail and (ii) the natal nest is the nearest nest to which the newly budded nest is attached. We use these assumptions to categorize all the nests within a colony as either: newly founded, founders (those from which a new nest has been budded), or nonfounders (those from which a new nest has not been budded). In some cases, the nearest nest to a newly founded nest was another newly founded nest. As the order of foundation cannot be inferred, the nearest established (i.e., not newly founded) nest was characterized as a possible founder. Newly founded nests can be either foraging or nonforaging and are founded both on and off existing trails (Ellis & Robinson, 2015). In addition, there is no seasonal effect of nest foundation within the time periods of our study: Nests are equally likely to be founded in spring and summer (Ellis & Robinson, 2015).

We used general linear mixed effect models (GLMMs) to analyze how budding relates to various nest, nest within the network, and colony attributes. In the GLMMs, founder status (i.e., founder, nonfounder, or possible founder) was used as the response variable with the variable(s) of interest as the fixed effect. Colony, nest ID, and season were included as random effects. Further details of the tests are given in the supporting information; the superscript in the text refers to the row of the table (Appendix S2). All GLMMs used a binomial error structure and a logit link function. We tested significance using a chi‐squared analysis of deviance (AoD) which compares the full model to a null model without the fixed effect. If the null model and full model are significantly different, it indicates that the fixed effect has a significant impact in explaining the data. Here we report the results of the AoD. GLMMs were performed in R using the “lme4” package (R Development Core Team R, 2011).

## 3 Results

### 3.1 Nest survival

**Figure 2:**
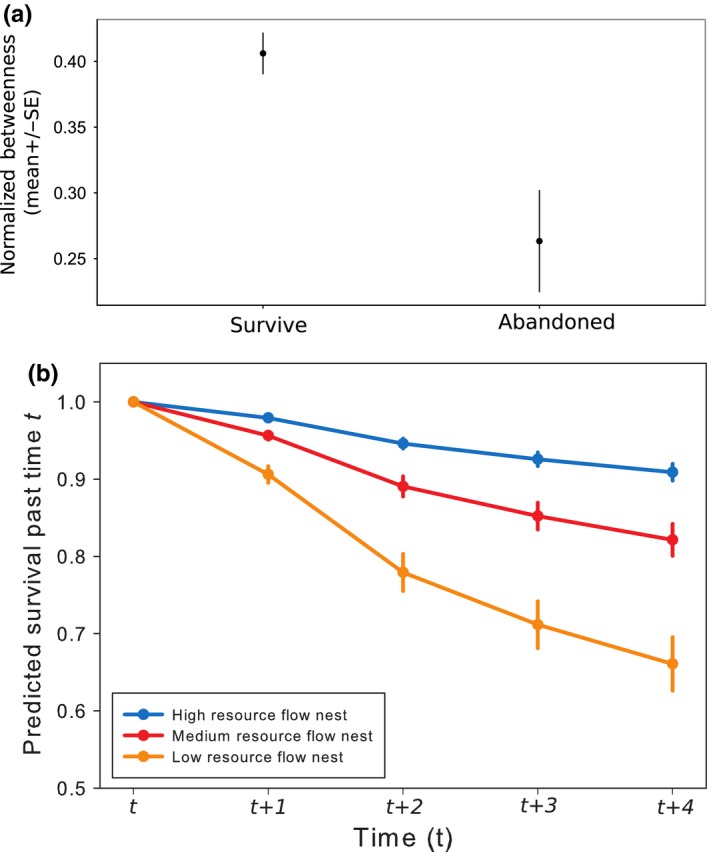
Nest survival depends on the flow (measured as normalized betweeness) of resources. (a) The mean (±standard error) normalized betweenness (potential resource flow) of nests which survive to the next time point, and the nests which are abandoned before the next time point. (b) Survival of nests predicted by the extended‐cox PH survival model. Curves represent how the survival of nests with a defined (and unchanging) resource flow is predicted to change with time. A high resource flow nest is a nest with a normalized betweeness of 0.9, medium resource flow nest is a nest with a normalized betweeness of 0.5, and a low resource flow nest is a nest with a normalized betweeness of 0.1. Curves are calculated using the Kaplan–Meier method. The error (SE) is the difference between survival in different colonies. Each point represents the survival of a nest at *t+x* time points after the nest is founded; four is the maximum time points after foundation as our study only covered five time points

The position of a nest within the network is a key predictor of its survival. Nests with a higher normalized betweeness are significantly more likely to survive than nests with a lower normalized betweeness (Cox PH: z = −3.8, *n* = 558, *p* = .0002; Figure 2). This relationship between normalized betweeness and survival is robust even when nest size is introduced into the survival model. Larger nests are significantly more likely to survive than smaller nests (Cox PH, z = −2.7, *n* = 558, *p* = .0026), but when included in the same model as an additional fixed effect, nests with a higher normalized betweeness are still significantly more likely to survive than nests with a lower normalized betweeness (Cox PH: normalized betweeness: z = −2.06, *n* = 558, *p* = .0146; nest size: z = −2.21, *n* = 558, *p* = .0198).

The survival of internest trails is, similarly, predicted by the position within the nest network of the nests they connect. Internest trails connect two nests; each nest has a value of normalized betweeness. The lower of these two normalized betweeness values is significantly related to the survival of the internest trail (Cox PH: z = −1.30, *n* = 476, *p* = .0373). The relationship is negative: A trail associated with a nest with a low normalized betweeness is less likely to survive than a trail associated with a nest with a high normalized betweeness. Nests with a high normalized betweeness are more likely to survive than nests with a low normalized betweeness (above) which may explain the negative relationship between trail survival and nest normalized betweeness. Trails associated with a nest with low normalized betweeness may be less likely to survive because the nest is less likely to survive, rather than due to the position of the trail within the network.

We found no relationships between survival and colony‐level effects. Nest survival is not significantly related to colony worker:foraging ratio (Cox PH: z = 0.24, *n* = 558, *p* = .3739), even when nest size is also included in the survival model (Cox PH: z = 0.38, *n* = 558, *p* = .3485). Similarly, trail survival is not significantly related to either trail betweeness (Cox PH: z = −1.68, *n* = 476, *p* = .0646) or colony worker:foraging ratio (Cox PH: z = −1.37, *n* = 476, *p* = .1016).

Attributes of the nests and trails can also influence their survival. Larger nests are significantly more likely to survive than smaller nests (Cox PH, z = −2.7, *n* = 558, *p* = .0026). However, the other nest attributes we measured do not affect survival. The survival of a given nest is not significantly affected by either the distance from the nest to the nearest tree (Cox PH: z = −1.24, *n* = 581, *p* = .1017) or the canopy cover over the nest (Cox PH: z = −0.17, *n* = 563, *p* = .3798). Internest trails with a high ant traffic were significantly more likely to survive than trails with low ant traffic (Cox PH: z = −2.4, *n* = 476, *p* = .0042). However, there is no significant relationship between trail weight (which is adjusted for the sizes of the connected nests) and trail survival (Cox PH: z = −0.59, *n* = 476, *p* = .2699).

### 3.2 Nest budding

**Figure 3:**
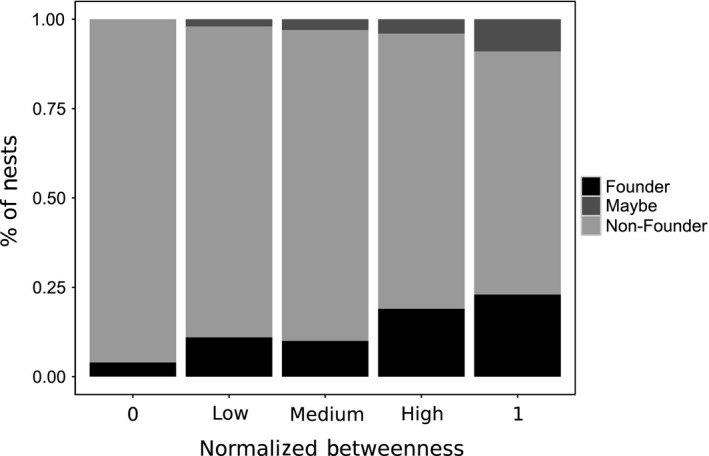
Nests with a higher normalized betweeness are more likely to act as founders of new nests than nests with a lower normalized betweeness (AoD: χ2 = 9.7, *df* = 2, *p *=* *.008). For the figure, betweeness is categorized as 0, 1, low (<0.25), medium (0.25–0.75), or high (>0.75)

Nests from which new nests have been founded (founders) have a significantly higher normalized betweeness than those from which no new nests have been founded (nonfounders) (AoD^1^: χ^2^ = 12.4, *df* = 1, *p* < .001; Figure 3). Nests often change in normalized betweeness between two time points. Founder nests have a significantly greater increase in normalized betweeness over the period which the new nest was founded than nonfounder nests (AoD^2^: χ^2^=14.7, *df* = 1, *p* < .0001).

Nests in colonies with a low worker:foraging ratio (i.e., a high foraging effort per worker) are not significantly more likely to be founders than nests in colonies with a high worker:foraging ratio (AoD^3^: χ^2^ = 0.15, *df* = 1, *p* = .70). Similarly, nests in colonies which have a lowered worker:foraging ratio (i.e., an increase in foraging effort per worker) are not significantly more likely to be founders than nest in colonies with a raised worker:foraging ratio (AoD^4^: χ^2^ = 0.04, *df* = 1, *p* = .80).

Nest attributes do not predict whether the nest has acted as a founder. Founder nests are not significantly larger than nonfounder nests (AoD^5^: χ^2^ = 0.20, *df* = 1, *p* = .65). Similarly, nests which had a greater increase in size are not significantly more likely to be founders than nests which have had a lower increase or a decrease in size (AoD^.6^: χ^2^ = 0.03, *df* = 2, *p* = .87). Founder nests are neither significantly closer to trees (AoD^7^: χ^2^ = 0.19, *df* = 1, *p* = .66), nor have a significantly lower canopy cover nest (AoD^8^: χ^2^ = 0.78, *df* = 1, *p* = .38) than nonfounder nests. Nests are not significantly more likely to act as founders in spring than in summer (AoD^9^: χ^2^ = 0.16, *df* = 1, *p* = .69).

We also investigated how the population change of nests is affected by nest attributes, nest position, and colony attributes. We found no significant effects (results reported in Appendix S3).

## 4 Discussion

In this study, we found that the position of a nest within the network of polydomous *Formica lugubris* colonies has important ecological consequences for that nest and the structure and integration of the colony. Nests with a higher flow of resources, even if this comes indirectly via other nests, have an increased chance of surviving and founding new nests than nests with a lower flow of resources; nest size is also accounted for and does not eliminate this effect. Distance to the nearest foraging tree does not affect nest survival. Resource flow through a nest depends on its connections to the other nests and how it fits into the broader structure of the network. The survival and budding of a nest is dependent on its relationship with other nests and the wider pattern of interaction between the nests in the polydomous colony. Our results show that, despite being spatially separated, the interconnected nests of a polydomous colony can be considered a single ecological unit, at least in terms of resource acquisition. We also demonstrate that dynamic network position can have important ecological consequences.

The view of the nests of polydomous wood ant colonies as forming a single ecological unit, supported by our results, suggests that the factors influencing the fitness of individuals in a given nest are likely to be strongly linked to the fitness of individuals in other nests. The ability of a nest to survive and bud depends, in part, on its position in the colony nest network. This dependence shows that the resource movement through the colony has an important ecological influence. Changes in the environment near any given nest have the potential to affect the survival and budding of nests throughout the network. However, it is important to note that the survival and reproduction of nests are driven by proximate process, namely the access of nests via the workers within them, to resources. This finding supports other work that has detected no evidence for top‐down, colony‐level effects on the structure of polydomous colonies (Ellis et al., 2014). The extent to which nests can be considered as part of the same colony, super‐colony, or super‐organism is an important consideration when assessing, for example, the level at which selection acts in a colony (Helanterä, Strassmann, Carrillo, & Queller, 2009; Kennedy, Uller, & Helanterä, 2014; Moffett, 2012).

Resources are often distributed heterogeneously in the environment; polydomy may be a way to more efficiently exploit these dispersed resources (Cook et al., 2013; Holway & Case, 2000; Lanan et al., 2011; Schmolke, 2009). The nest and foraging network of polydomous colonies can be viewed as a transportation network to move resources from food sources to the nests and then between nests (Cook et al., 2014; Latty et al., 2011). Transport efficiency refers to the ease with which resources can flow through a network. In the polydomous nest system, nests with a high resource flow are at points in the network important for colony‐level resource redistribution and therefore colony‐level transport efficiency (e.g., Croft et al., 2008; Perna & Latty, 2014). If network transport efficiency is being retained within a colony, nests and trails with a higher resource flow, and therefore greater importance for efficiency, may be more likely to survive than those with a lower betweeness. We found that nests with a high resource flow are more likely to survive than nests with a lower betweeness. However, trails with a higher resource flow are not more likely to survive than trails with a lower betweeness. Efficient transport structures are therefore not preferentially being retained in the nest network. Additionally, the process of nest foundation will also degrade efficient transport structures. As new nests are founded by nests with a high transport value (a high flow of resources), this will alter the structure of the colony around that nest. This establishment of new nests and trails will change the previously existing, efficient, transport structures. In a system which is under strong selective pressure for efficiency, it is expected that highly effective transport structures will be retained. As this is not the case in the red wood ant polydomous colonies, it may be that transport efficiency is not under strong selective pressure.

The flow of resources through a particular nest can change over time due to other nests in the network being gained and lost. The integrated nature of the system means that a given nest could maintain the same connections to neighboring nests and trees but still undergo a change in the amount of resources available to it (and therefore its chances of surviving and reproducing), due to nests being abandoned or founded elsewhere in the colony. Nests in unprofitable areas, and therefore with a low resource flow, are more likely to be abandoned than nests in profitable areas. These dynamics will result in the colony moving toward resources and away from unprofitable areas. For a spatially embedded network, such as a polydomous network, this movement is physical movement of nodes. In networks which are not spatially embedded, such as social networks, this process could result in a network clustering around certain nodes, for example individuals with information. The reverse could also occur; a network could cluster away from specific nodes, for example diseased individuals in a social network. These changes in the network structure are self‐organized, resulting from selective pressure based on an individual's position in the network.

The nest networks of polydomous ant colonies are, in some ways, analogous to the social networks of individual organisms. Like individuals, ant nests can survive and reproduce (in the sense of founding new nests). There are, however, crucial differences. For example, the death of an individual animal in a social network has direct fitness consequences. In contrast, although abandoning a nest will result in the loss of the resources invested in constructing the nest (which may be considerable), it is unlikely to result in the death of the ants in the nest; they will simply join other nests in the colony. Despite these important differences, polydomous ant colonies may be useful models of social networks. Similar to the ant nest networks, the position of an individual in a social network can have important consequences for their access to, for example, information (e.g., Blonder & Dornhaus, 2011; Farine, Aplin, Sheldon, & Hoppitt, 2015) and disease (e.g., Cross et al., 2004; Otterstatter & Thomson, 2007). However, linking these network position effects to the life history of individuals is challenging, due to the difficulties in collecting sufficiently high‐quality temporal data to allow the networks to be examined dynamically (Croft et al., 2008, 2011; Kurvers et al., 2014; Whitehead, 2008). Using the polydomous nest networks, we have demonstrated that network position can have an important influence on the survival, population change, and budding in a dynamic system. This provides a useful basis for examining the importance of network position in other biological systems such as social systems.

The network dynamics observed in these polydomous colonies illustrate the potential feedback between the individual level and the system level in biological networks. The position of an individual within a biological system can affect that individual's exposure to, for example, food, mates, information, and disease (e.g., Aplin, Farine, Morand‐Ferron, & Sheldon, 2012; Christley et al., 2005; Oh & Badyaev, 2010). The structure of the network is, in turn, affected by the nodes within the network. For example, the overall pattern of interactions between individuals in a system can be influenced by a variety of biotic and abiotic factors such as food availability, sex demographics, and season (Brent, MacLarnon, Platt, & Semple, 2013; Darden, James, Ramnarine, & Croft, 2009; Foster et al., 2012). The nests within polydomous colonies highlight how these effects can be reciprocal in a dynamic system. Differential survival and reproduction of nodes in a system will change the structure of the network as new nodes appear and others disappear. This will, in turn, change an individual's relative position within the network, altering its chances of surviving and reproducing. The network, therefore, will be continually restructuring, resulting in a dynamic system which is not stable through time. Dynamic processes will react differently to static systems when facing ecological and environmental changes (Kurvers et al., 2014).

In conclusion, we found that the survival and budding of nests within polydomous *Formica lugubris* colonies are related to their position in the trail network. These results highlight how apparently disparate entities in a biological system can be integrated into a functional ecological unit. It also shows how indirect access to resources, through others in a resource exchange system, can have important ecological consequences.

## Conflict of Interest

None declared.

## Data Accessibility

Data are provided in the supplementary materials.
