# Supplementary material for: Ecological consequences of colony structure in dynamic ant nest networks
Source: Ecol Evol. 2017 Jan 24;7(4):1170–80. doi: 10.1002/ece3.2749 (PMC5306006; doi:10.1002/ece3.2749)
Supplement: Supplementary file 1 [file ECE3-7-1170-s001.pdf]

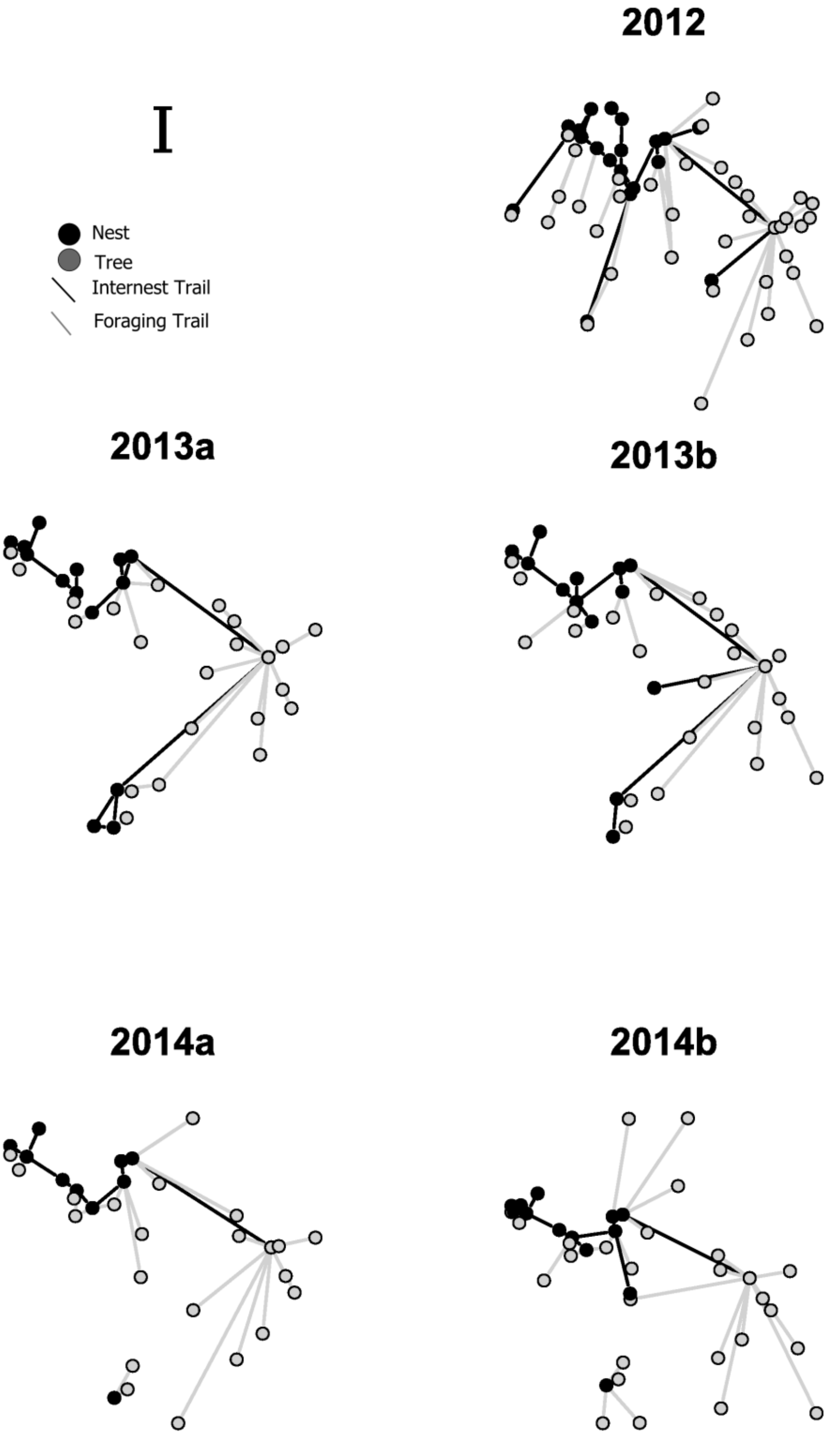

Nest
Tree
Internest Trail
Foraging Trail
2012
2013a
2013b
2014a
2014b

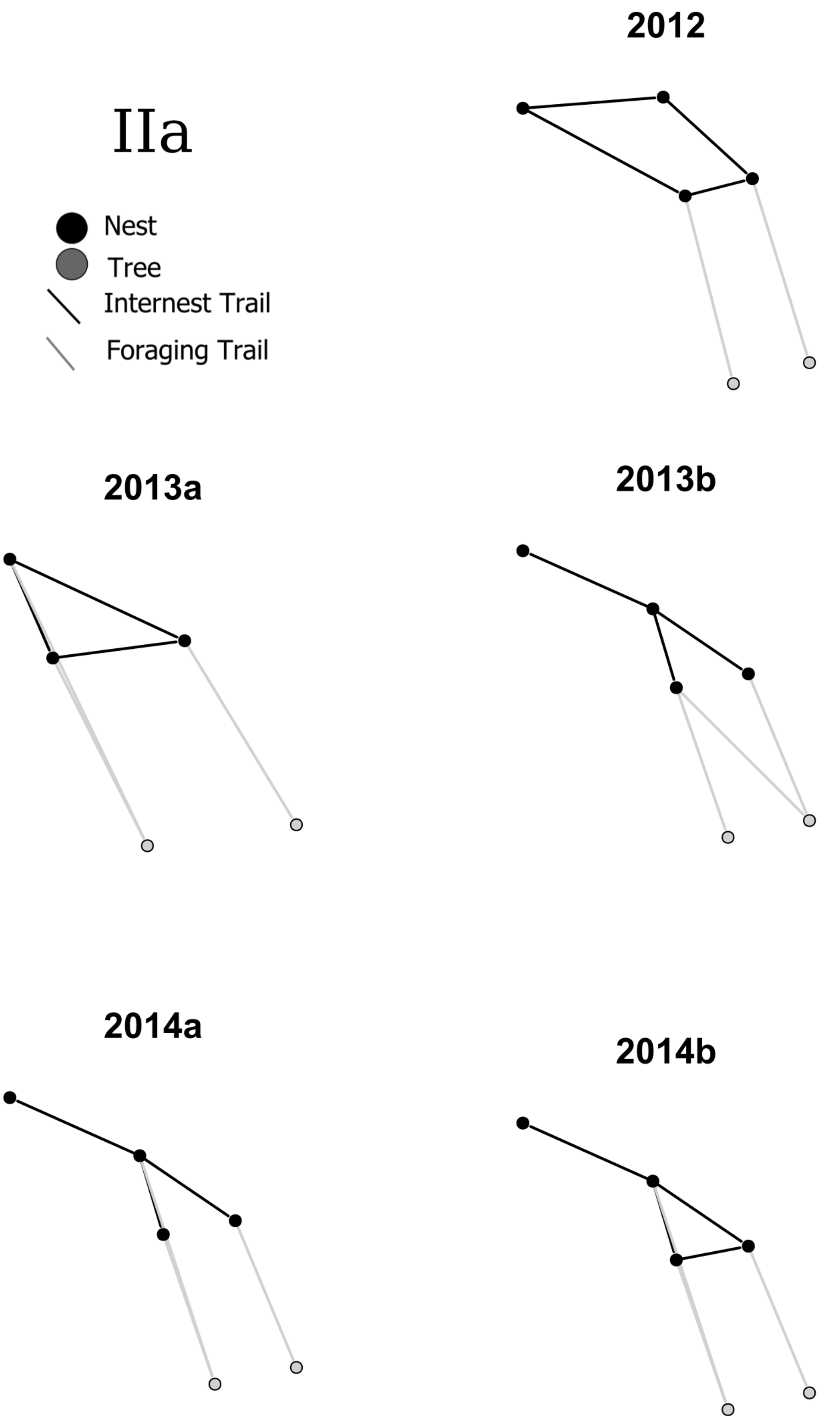

IIa
Nest
Tree
Internest Trail
Foraging Trail
2012
2013a
2013b
2014a
2014b

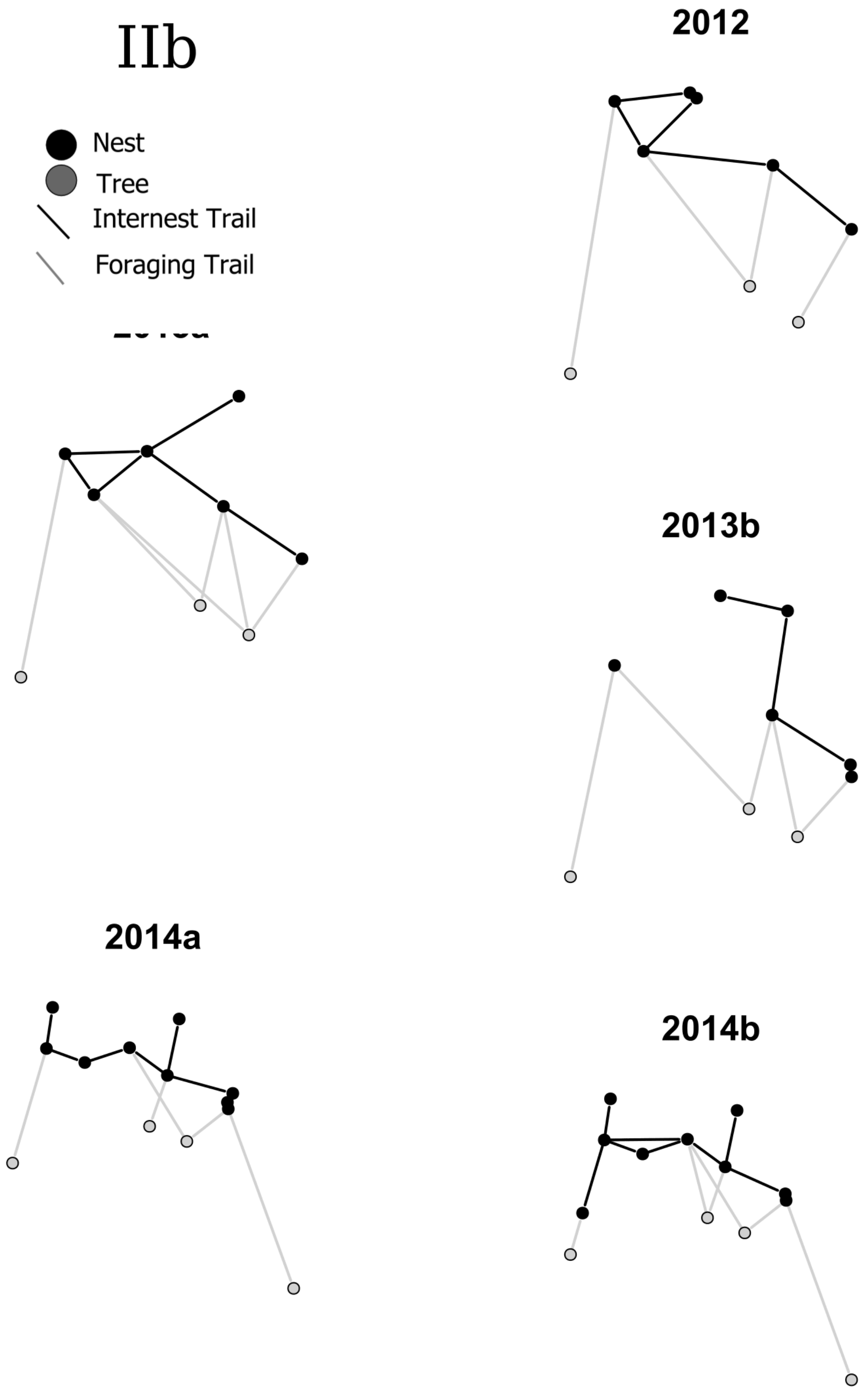
IIb
Nest
Tree
Internest Trail
Foraging Trail
2012
2013b
2014a
2014b

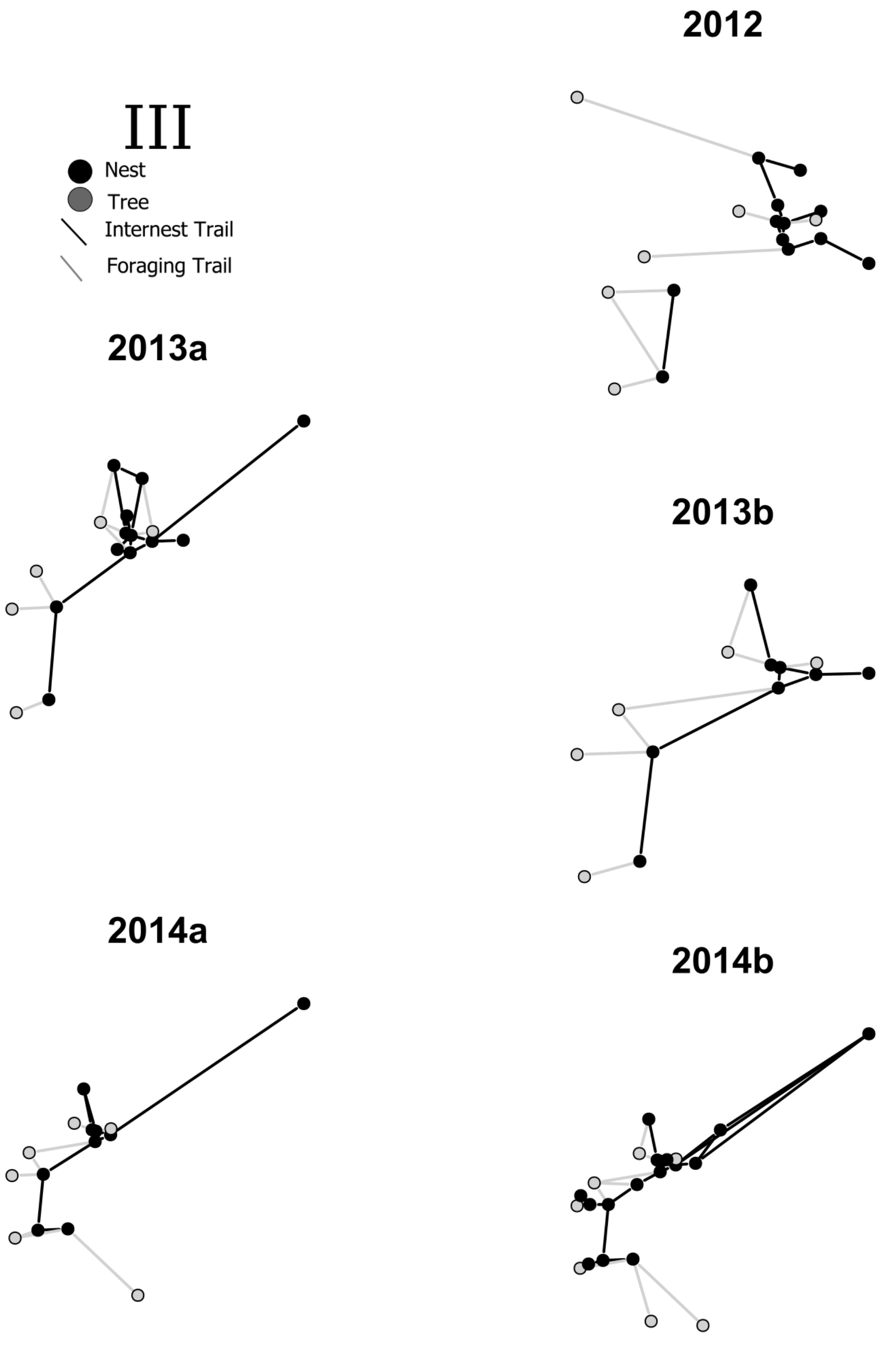
III
Nest
Tree
Internest Trail
Foraging Trail
2012
2013a
2013b
2014a
2014b

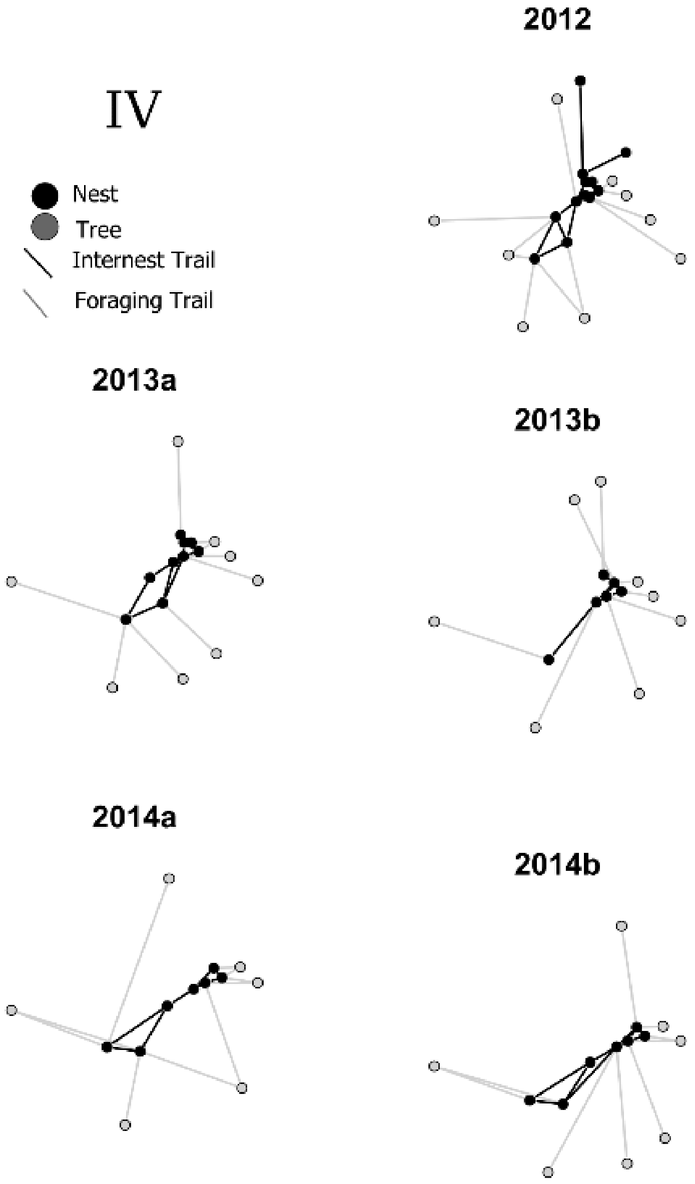

IV
Nest
Tree
Internest Trail
Foraging Trail
2012
2013a
2013b
2014a
2014b

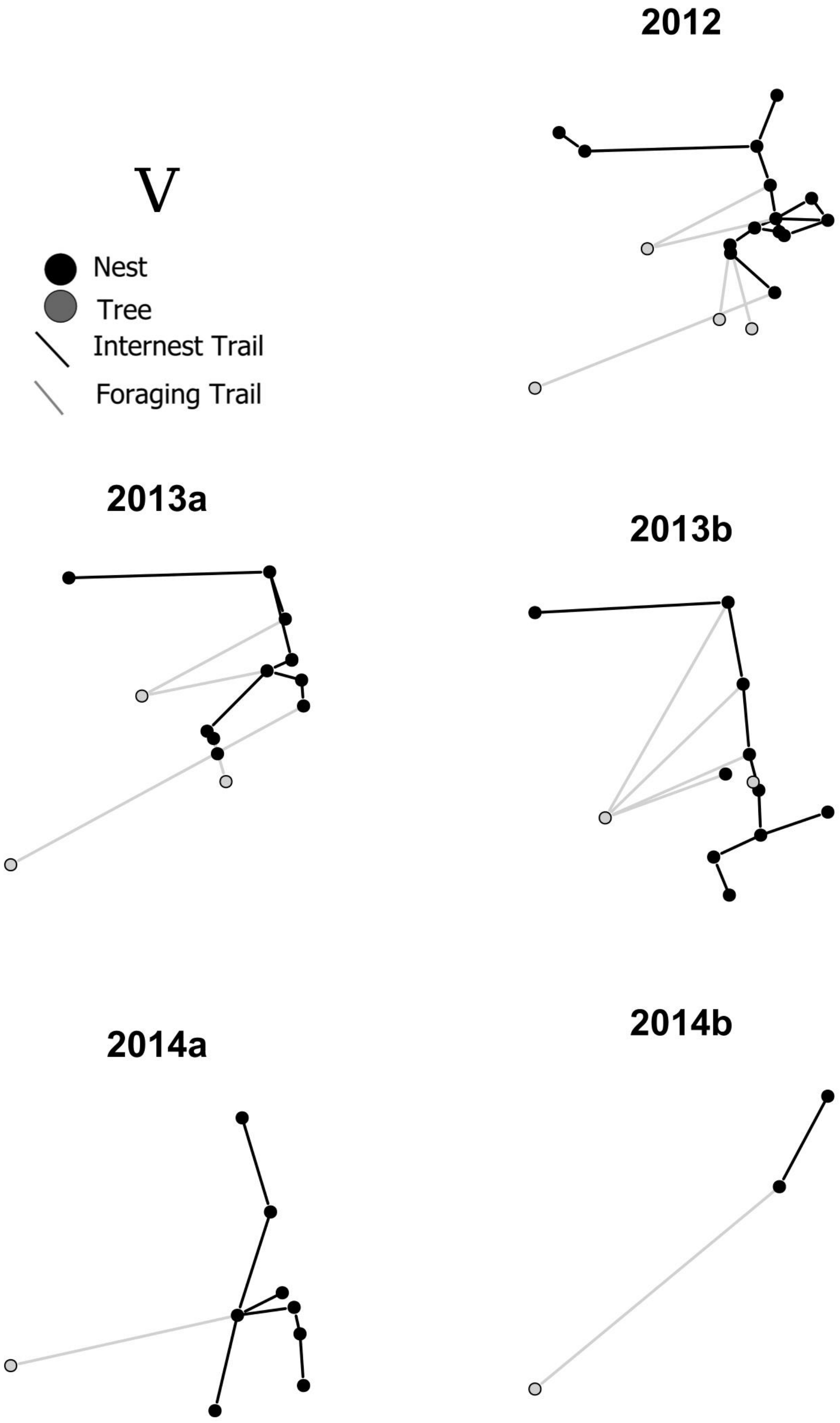
V
Nest
Tree
Internest Trail
Foraging Trail
2012
2013a
2013b
2014a
2014b

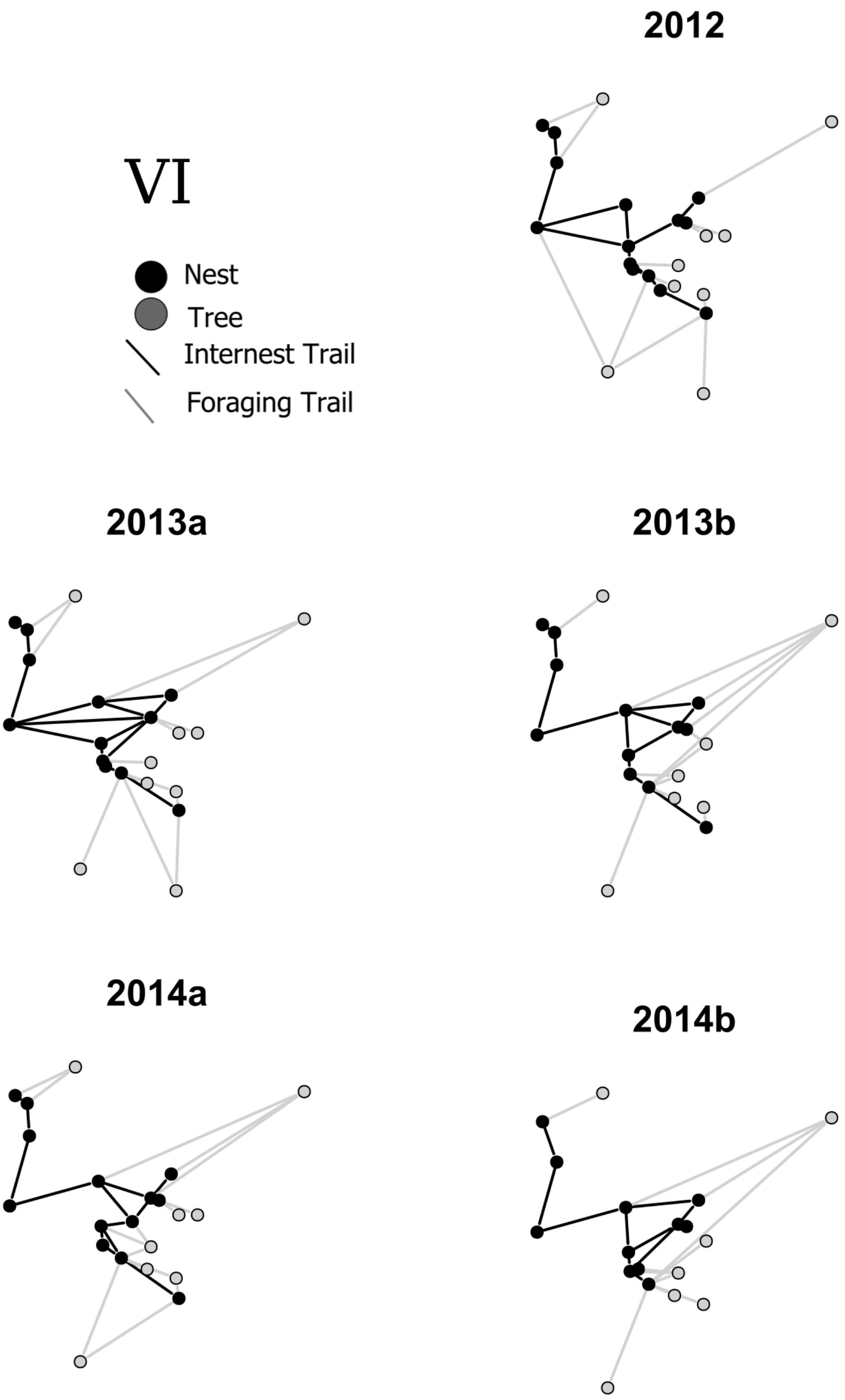

VI
Nest
Tree
Internest Trail
Foraging Trail
2012
2013a
2013b
2014a
2014b

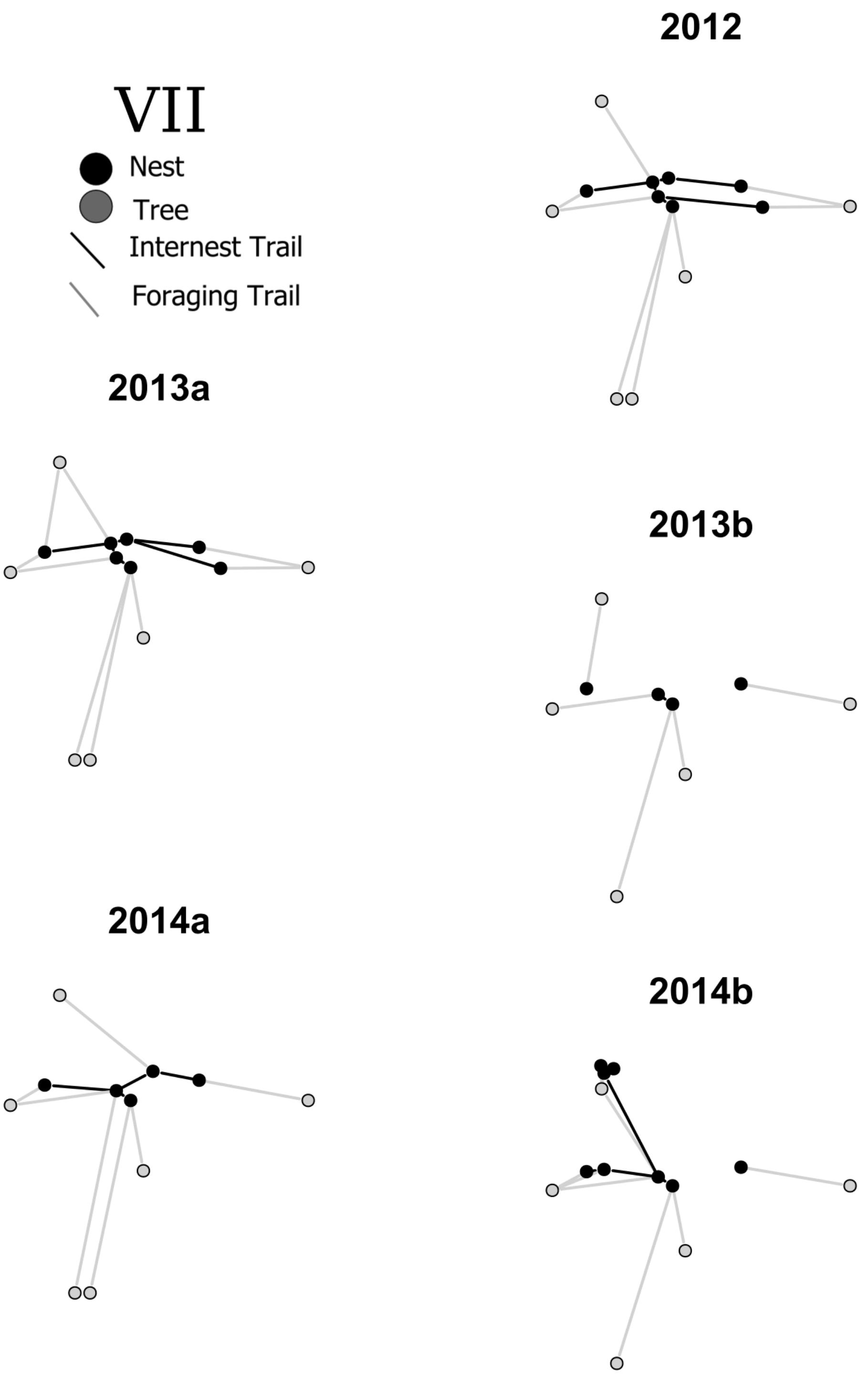

VII
Nest
Tree
Internest Trail
Foraging Trail
2012
2013a
2013b
2014a
2014b

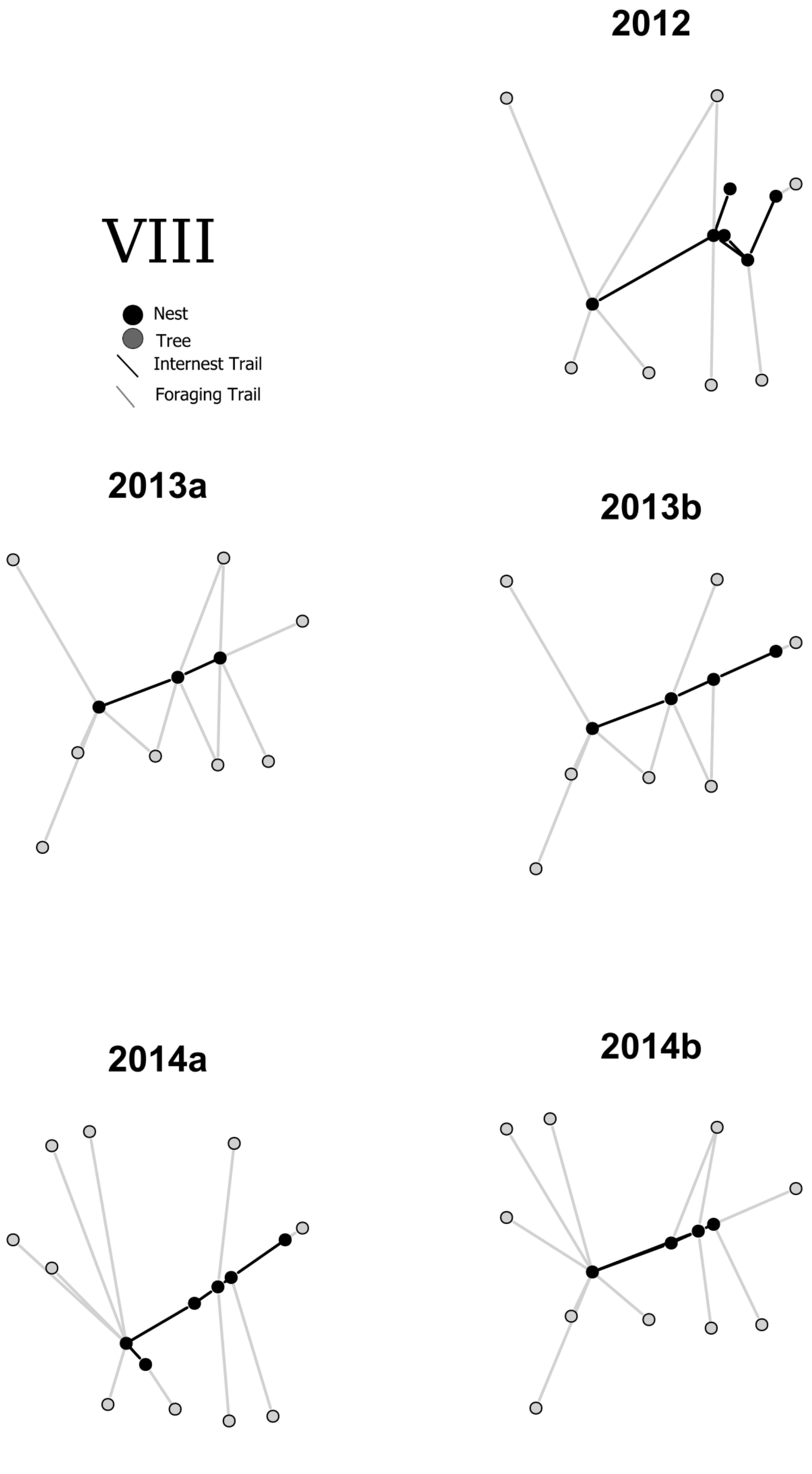
VIII
Nest
Tree
Internest Trail
Foraging Trail
2012
2013a
2013b
2014a
2014b

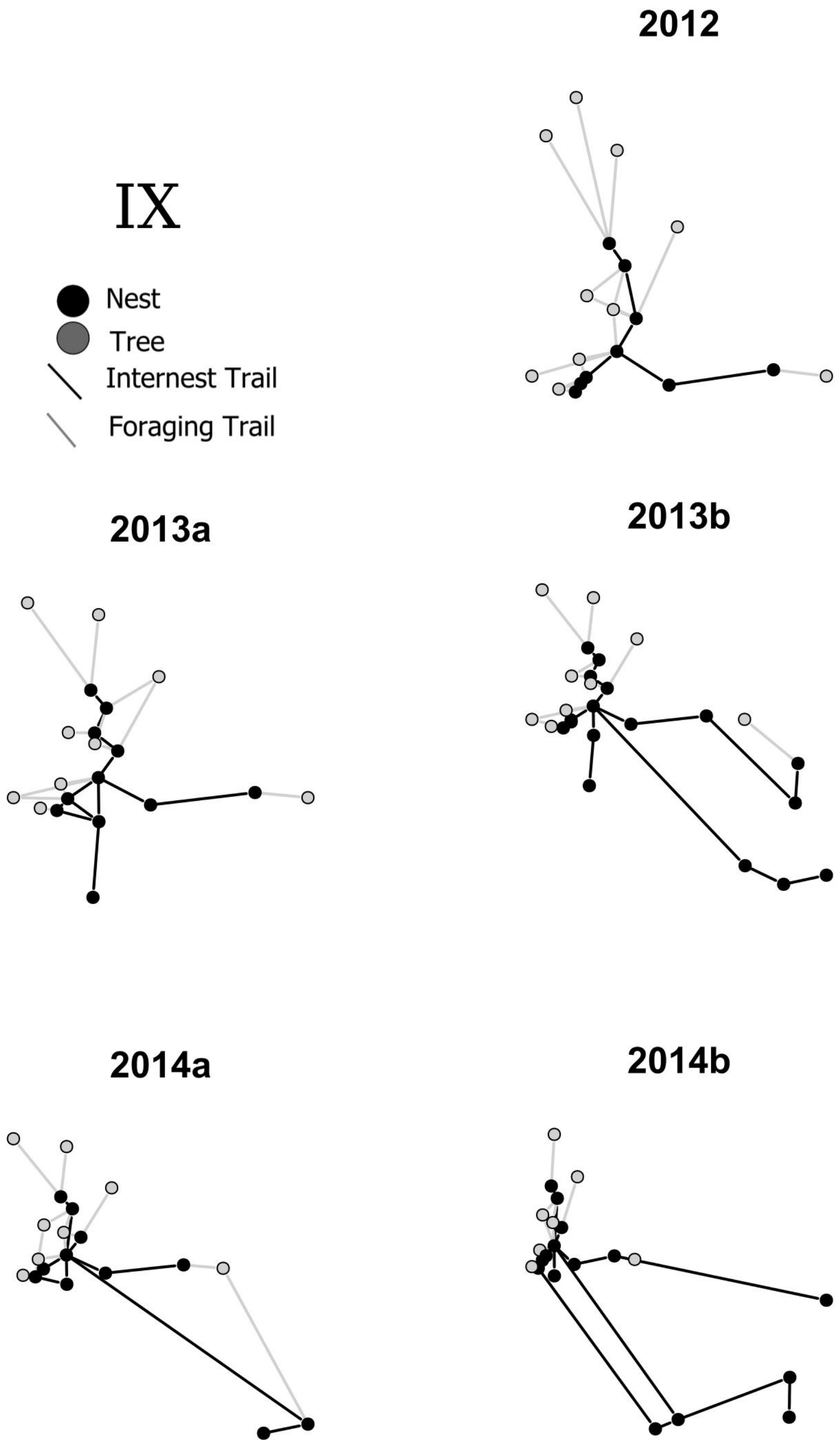
IX
Nest
Tree
Internest Trail
Foraging Trail
2012
2013a
2013b
2014a
2014b

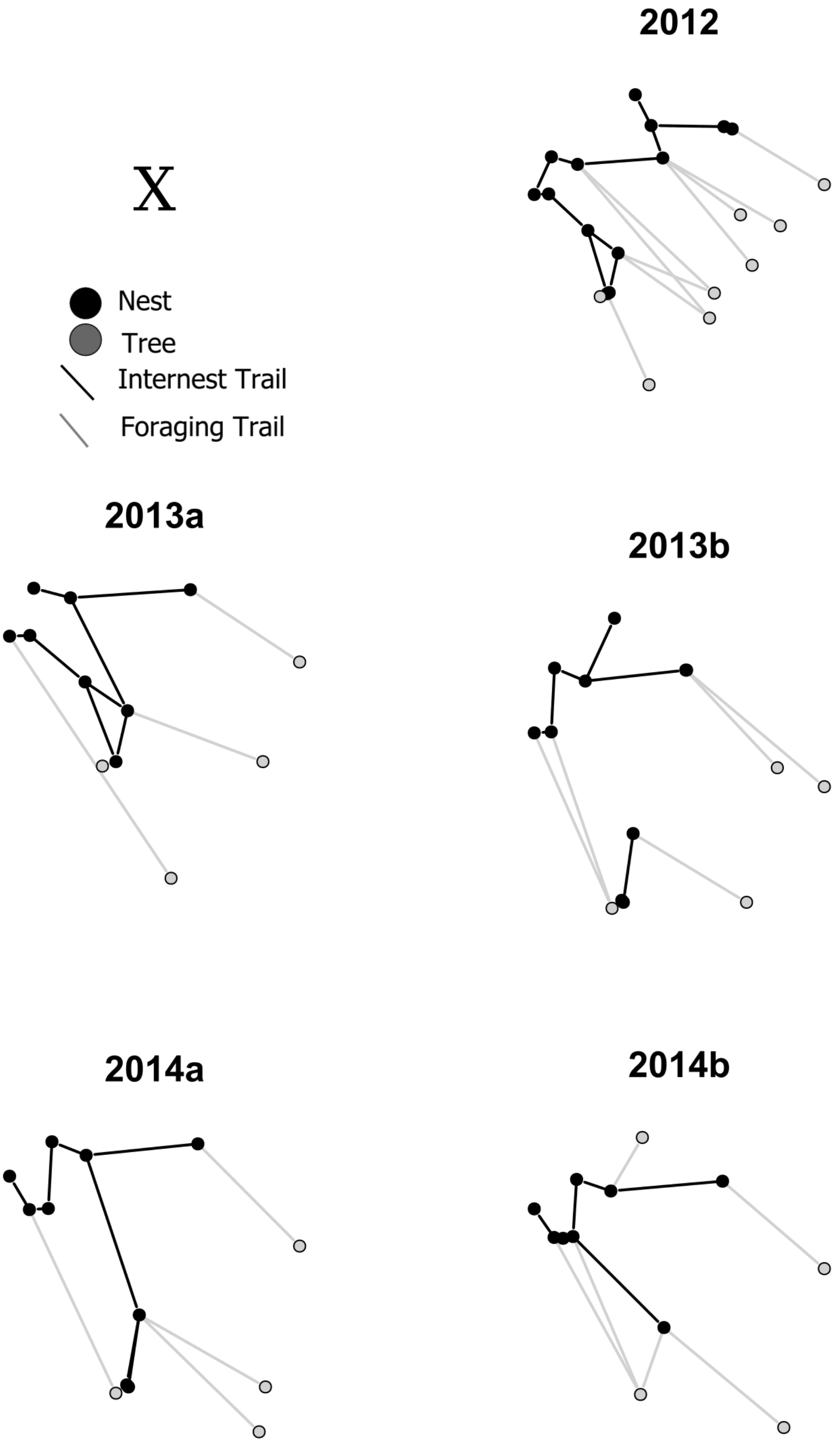
X
2012
Nest
Tree
Internest Trail
Foraging Trail
2013a
2013b
2014a
2014b

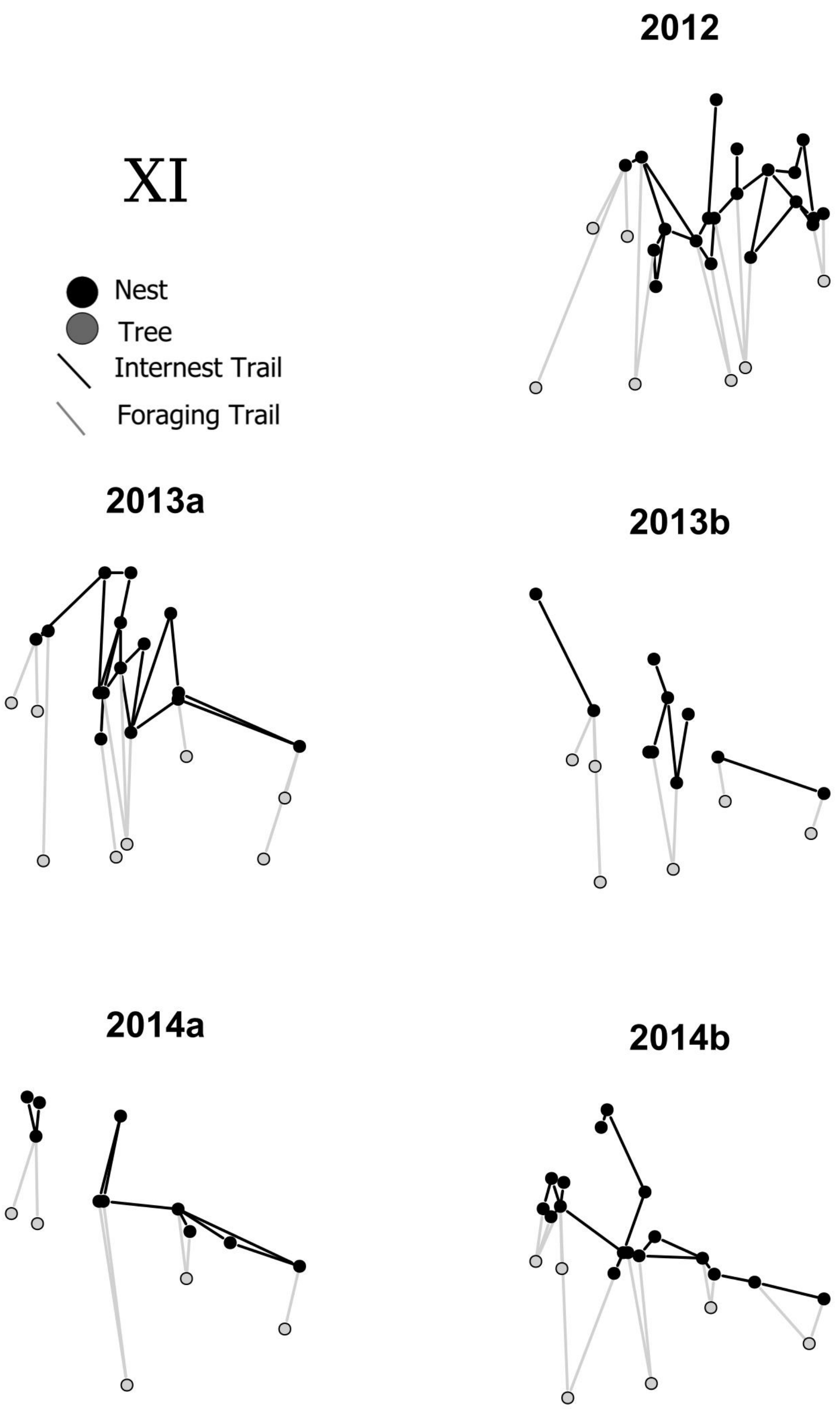
XI
Nest
Tree
Internest Trail
Foraging Trail
2012
2013a
2013b
2014a
2014b

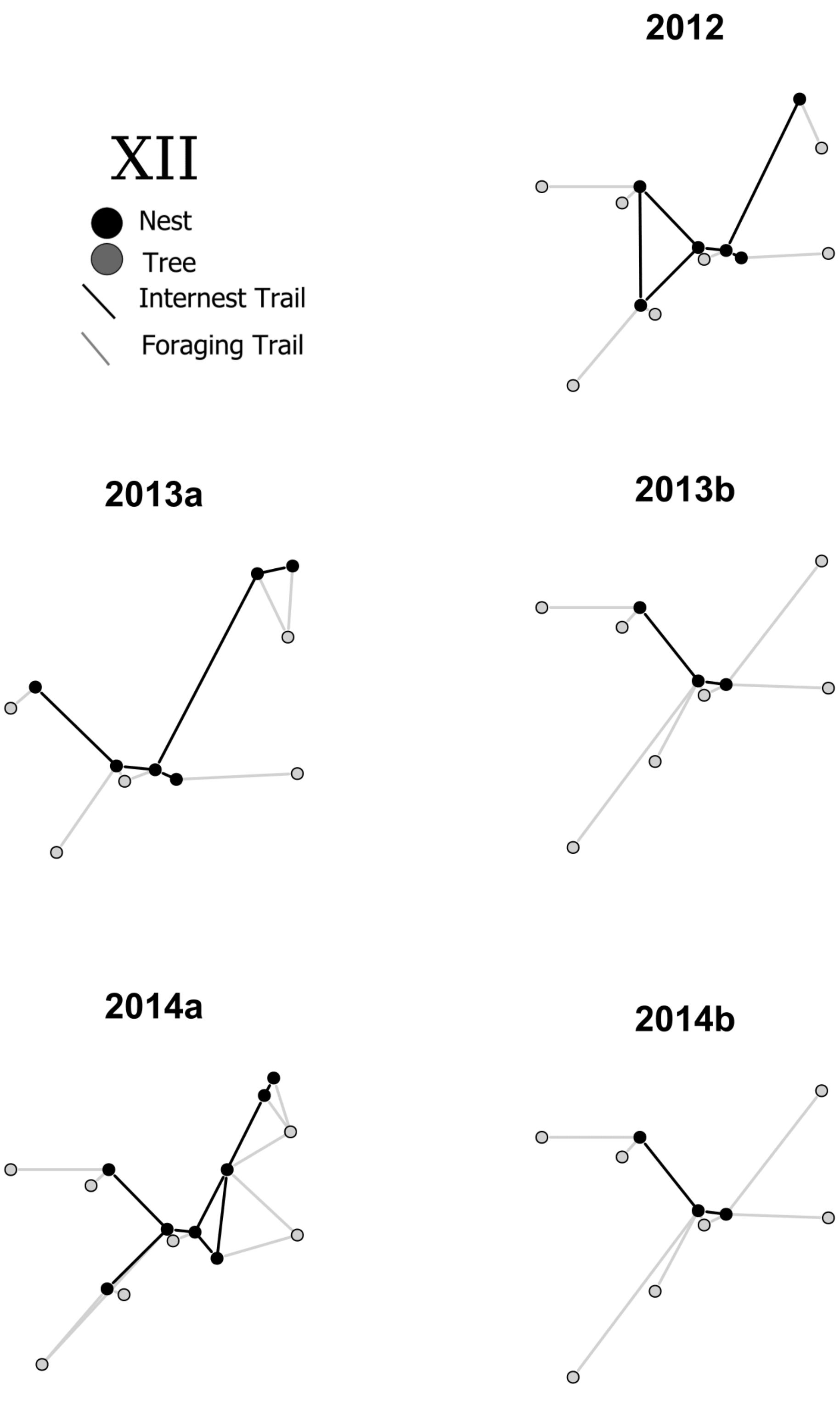
XII
Nest
Tree
Internest Trail
Foraging Trail
2012
2013a
2013b
2014a
2014b
