# Supplementary material for: Ecological consequences of colony structure in dynamic ant nest networks
Source: Ecol Evol. 2017 Jan 24;7(4):1170–80. doi: 10.1002/ece3.2749 (PMC5306006; doi:10.1002/ece3.2749)
Supplement: Supplementary file 2 [file ECE3-7-1170-s002.docx]

**Appendix B: Details of statistics**

Details of the statistics used in the study. # refers to the superscript number in the text. The Dependent variable, fixed effects and random effects describe the GLMM used, all used a binomial error structure. In all tests errors were heteroscedastic and were not overdispersed. χ^2^, *df* and *P* describe the results of an analysis of deviance, which compares the model to a null model which lacks the variable of interest.

| # | Dependent Variable | Fixed Effect(s) | Random Effect(s) | χ^2^ | *df* | *P* |
| --- | --- | --- | --- | --- | --- | --- |
| 1 | Founder or non-founder | Normalised Betweeness | Colony  Nest ID  Season | 12.4 | 1 | <0.001 |
| 2 | Founder or non-founder | Change in normalised betweeness | Colony  Nest ID | 14.7 | 1 | <0.001 |
| 3 | Founder or non-founder | Worker:foraging ratio | Colony  Nest ID  Season | 0.15 | 1 | 0.70 |
| 4 | Founder or non-founder | Proportional change in worker:foraging ratio | Colony  Nest ID | 0.04 | 1 | 0.8 |
| 5 | Founder or non-founder | Nest Size | Colony  Nest ID  Season | 0.2 | 1 | 0.65 |
| 6 | Founder or non-founder | Proportional change in nest size | Colony  Nest ID | 0.027 | 1 | 0.87 |
| 7 | Founder or non-founder | Linear distance to the nearest tree | Colony  Nest ID | 0.019 | 1 | 0.66 |
| 8 | Founder or non-founder | Percentage canopy cover | Colony  Nest ID | 0.78 | 1 | 0.38 |
| 9 | Founder or non-founder | Season | Colony  Nest ID | 0.16 | 1 | 0.69 |
| 10 | Proportional Change in nest size | Change in normalised betweeness | Colony  Nest ID  Season | 0.05 | 1 | 0.82 |
| 11 | Proportional Change in nest size | Proportional change in mean number of foragers per nest | Colony  Nest ID | 0.14 | 1 | 0.7 |
| 12 | Proportional Change in Nest size | Linear distance to the nearest tree | Colony  Nest ID | 0.0818 | 1 | 0.77 |
| 13 | Proportional change in nest size | Percentage canopy cover | Colony  Nest ID | 0.85 | 1 | 0.38 |
