# Supplementary material for: Ecological consequences of colony structure in dynamic ant nest networks
Source: Ecol Evol. 2017 Jan 24;7(4):1170–80. doi: 10.1002/ece3.2749 (PMC5306006; doi:10.1002/ece3.2749)
Supplement: Supplementary file 3 [file ECE3-7-1170-s003.docx]

**Methods**

We investigated the change in nest size (which can be positive or negative) over time by comparing the within nest population of a given nest at two time-points. We used GLMMs to investigate how the change in size related to other nest, nest-within-network and colony factors. In these analyses proportional change in nest size was used as the response variable, the factor of interest as the fixed effect and colony, nest ID and season as random effects (further details in appendix B). GLMMs used a Gaussian error structure and an identity link function. Analysis of deviance was used to test the significance of variables.

**Results**

Nests often changed considerably in size between time-points, ranging from an increase of over 6000% to a decrease of 99%. The median change in size was a decrease of 17% and the mean change in size was an increase of 91%. Change in nest size is significantly positively related to change in normalised betweeness (AoD^10^: χ^2^=0.05, df=1, p=0.82; figure 4). Nests with an increase normalised betweeness usually grew size, whereas nests with a decrease in normalised betweeness usually showed a reduction in size.

Worker:foraging ratio is calculated by dividing the total sizes of all the nests in the colony by the foraging effort of the colony. Due to the potentially confounding effect of using nest size to calculate both worker:foraging ratio and the change in nest size we use an alternative measure of colony foraging effort. We use the mean number of foragers per nest as a substitute colony-level attribute. There is no significant relationship between the change in nest size and the change in mean number of foragers per nest (AoD^11^: χ^2^=0.14, df=1, p=0.70).

Nests which are nearer to trees do not show a significantly different change in size than those which are further from trees (AoD^12^: χ^2^=0.08, df=1, p=0.7748). Similarly, change in nest size is not significantly related to the canopy cover over the nest (AoD^13^: χ^2^=0.85, df=1, p=0.36).

**Conclusion**

There is no significant effect of nest attributes, nest-position or colony-attributes on the size change of *Formica lugubris* nests.
