# Supplementary material for: Ecological consequences of colony structure in dynamic ant nest networks
Source: Ecol Evol. 2017 Jan 24;7(4):1170–80. doi: 10.1002/ece3.2749 (PMC5306006; doi:10.1002/ece3.2749)
Supplement: Supplementary file 4 [file ECE3-7-1170-s004.pdf]

Supplementary Information.

<u>Key</u>

Sizes: Nest population as number of workers.

Canopy: % of the area above the nest obscured by canopy.

Tree species: Species of tree. SP= Scots Pine

TotalForageWeight: The total weight of foraging trails from a nest. Calculated as outlined in the text.

Norm.between: The normalised betweenness (Resource flow) through a nest.

I.2012

	node.id	type	sizes	canopy	treespecies	totalforageweight	norm.between
V1	1	nest	6695	31.41	NA	550	0.0591341077
V2	2	nest	3753	42.59	NA	5	0.2291446674
V3	3	nest	6557	38.73	NA	0	0
V4	4	nest	6219	33.91	NA	0	0.2745512144
V5	5	nest	7247	20.93	NA	0	0
V6	6	nest	18299	32.87	NA	4344	0.5216473073
V7	7	nest	26490	38.03	NA	740	0.5818373812
V8	8	nest	10059	39.66	NA	0	0.6040126716
V9	9	nest	74951	58.39	NA	3694.74	0.8437170011
V10	10	nest	625	33.67	NA	0	0.116156283
V11	11	nest	5834	11.75	NA	0	0.0591341077
V12	12	nest	1303	3.58	NA	0	0
V13	13	nest	2539	60.17	NA	247.85	0.1172122492
V14	14	nest	21489	54.14	NA	721.75	0.227032735
V15	15	nest	2071	53.83	NA	0	0.9503695882
V16	16	nest	57120	19.71	NA	0	0.8511087645
V17	17	nest	48199	48.26	NA	5600.66	0.0887011616
V18	18	nest	40626	18.29	NA	5707.9	1
V19	19	nest	53463	69.08	NA	2	0.0591341077
V20	20	nest	1791627	63.04	NA	82716.79	0.9809926082
V21	21	nest	4001	48.88	NA	399.6	0.0591341077
V26	26	tree	NA	NA	SP	0	0
V27	27	tree	NA	NA	oak	0	0
V28	28	tree	NA	NA	SP	0	0
V29	29	tree	NA	NA	SP	0	0
V30	30	tree	NA	NA	oak	0	0
V31	31	tree	NA	NA	SP	0	0
V32	32	tree	NA	NA	SP	0	0
V33	33	tree	NA	NA	silver birch	0	0
V34	34	tree	NA	NA	SP	0	0
V35	35	tree	NA	NA	SP	0	0
V36	36	tree	NA	NA	sycamore	0	0
V37	37	tree	NA	NA	sycamore	0	0
V38	38	tree	NA	NA	SP	0	0
V39	39	tree	NA	NA	silver birch	0	0
V40	40	tree	NA	NA	SP	0	0
V41	41	tree	NA	NA	SP	0	0
V42	42	tree	NA	NA	SP	0	0
V43	43	tree	NA	NA	SP	0	0
V44	44	tree	NA	NA	SP	0	0
V45	45	tree	NA	NA	SP	0	0
V46	46	tree	NA	NA	SP	0	0
V47	47	tree	NA	NA	SP	0	0
V48	48	tree	NA	NA	SP	0	0
V49	49	tree	NA	NA	SP	0	0
V50	50	tree	NA	NA	SP	0	0
V51	51	tree	NA	NA	SP	0	0
V52	52	tree	NA	NA	oak	0	0
V53	53	tree	NA	NA	oak	0	0
V54	54	tree	NA	NA	sycamore	0	0
V55	55	tree	NA	NA	oak	0	0
V56	56	tree	NA	NA	SP	0	0

V57	57	tree	NA	NA	SP	0	0
V58	58	tree	NA	NA	SP	0	0
V59	59	tree	NA	NA	SP	0	0
V60	60	tree	NA	NA	SP	0	0
V61	61	tree	NA	NA	SP	0	0
V62	62	tree	NA	NA	SP	0	0

	node.id	type	sizes	canopy	treespecies	totalforageweight	norm.between
V2	2	nest	3306	55.19	NA	0.5	0.031358885
V3	3	nest	3786	31.18	NA	0	0.0557491289
V4	4	nest	2443	51.48	NA	0	0.0731707317
V5	5	nest	22178	6.29	NA	0	0
V6	6	nest	2017	46.56	NA	699.3	0.1149825784
V8	8	nest	1575	52.26	NA	0	0.0731707317
V9	9	nest	8929	41.18	NA	1770	0.0592334495
V10	10	nest	1358	18.35	NA	0	0
V15	15	nest	11095	62.3	NA	66.99	0.0871080139
V16	16	nest	6089	1.69	NA	0	0
V17	17	nest	2382	36.51	NA	1586.31	0.3240418118
V18	18	nest	11233	19.68	NA	5600	0.4668989547
V20	20	nest	933725	50.03	NA	14065.65	1
V22	22	nest	3306	7.2	NA	0	0
V23	23	nest	11233	58.12	NA	643	0.3240418118
V24	24	nest	44881	58.99	NA	96.48	0.0871080139
V27	27	tree	NA	NA	SP	0	0
V28	28	tree	NA	NA	SP	0	0
V32	32	tree	NA	NA	SP	0	0
V34	34	tree	NA	NA	SP	0	0
V37	37	tree	NA	NA	SP	0	0
V38	38	tree	NA	NA	SP	0	0
V42	42	tree	NA	NA	sycamore	0	0
V44	44	tree	NA	NA	SP	0	0
V45	45	tree	NA	NA	silver birch	0	0
V46	46	tree	NA	NA	SP	0	0
V47	47	tree	NA	NA	SP	0	0
V48	48	tree	NA	NA	SP	0	0
V49	49	tree	NA	NA	SP	0	0
V51	51	tree	NA	NA	sycamore	0	0
V54	54	tree	NA	NA	SP	0	0
V56	56	tree	NA	NA	SP	0	0
V57	57	tree	NA	NA	SP	0	0
V59	59	tree	NA	NA	SP	0	0
V60	60	tree	NA	NA	SP	0	0
V63	63	tree	NA	NA	SP	0	0
V64	64	tree	NA	NA	sycamore	0	0
V65	65	tree	NA	NA	SP	0	0

	node.id	type	sizes	canopy	treespecies	totalforageweight	norm.between
V2	2	nest	6798	47.42	NA	1	0.0759753593
V3	3	nest	10199	39.61	NA	0	0.1478439425
V5	5	nest	36188	21.58	NA	0	0
V6	6	nest	25586	10.12	NA	525	0.3531827515
V8	8	nest	2751	54.53	NA	0	0.3942505133
V9	9	nest	84505	38.34	NA	4335	0.7310061602
V10	10	nest	277	33.71	NA	0	0
V15	15	nest	19045	73.25	NA	101.5	0.0759753593
V16	16	nest	58854	24.93	NA	0	0.8028747433
V17	17	nest	19045	48.66	NA	3286.71	0.1498973306
V18	18	nest	56868	19.58	NA	6324.05	0.8501026694
V20	20	nest	570248	61.76	NA	36043.37	1
V23	23	nest	1752	54.96	NA	6330	0.2197125257
V24	24	nest	157142	67.1	NA	240.48	0.0759753593
V25	25	nest	2284	62.94	NA	0	0
V27	27	tree	NA	NA	SP	0	0
V28	28	tree	NA	NA	SP	0	0
V31	31	tree	NA	NA	SP	0	0
V32	32	tree	NA	NA	sycamore	0	0
V34	34	tree	NA	NA	SP	0	0
V37	37	tree	NA	NA	SP	0	0
V38	38	tree	NA	NA	SP	0	0
V42	42	tree	NA	NA	SP	0	0
V43	43	tree	NA	NA	silver birch	0	0
V44	44	tree	NA	NA	SP	0	0
V45	45	tree	NA	NA	SP	0	0
V46	46	tree	NA	NA	sycamore	0	0
V47	47	tree	NA	NA	SP	0	0
V48	48	tree	NA	NA	SP	0	0
V49	49	tree	NA	NA	silver birch	0	0
V51	51	tree	NA	NA	SP	0	0
V56	56	tree	NA	NA	sycamore	0	0
V57	57	tree	NA	NA	oak	0	0
V58	58	tree	NA	NA	SP	0	0
V59	59	tree	NA	NA	SP	0	0
V60	60	tree	NA	NA	SP	0	0
V63	63	tree	NA	NA	SP	0	0
V64	64	tree	NA	NA	SP	0	0
V65	65	tree	NA	NA	SP	0	0

	node.id	type	sizes	canopy	treespecies	totalforageweight	norm.between
V3	3	nest	4807	28.35	NA	190	0.1138211382
V5	5	nest	69831	12.56	NA	0	0
V6	6	nest	34268	40.33	NA	597.5	0.4268292683
V8	8	nest	5317	51.15	NA	0	0.487804878
V9	9	nest	14373	56.96	NA	1910.5	0.6504065041
V15	15	nest	11161	48.2	NA	845.95	0.8089430894
V16	16	nest	6208	26.58	NA	0	0.8536585366
V17	17	nest	14269	42.13	NA	4212.34	0.9390243902
V18	18	nest	7362	22.32	NA	12132.78	1
V20	20	nest	245353	61.91	NA	34564.41	0.9552845528
V24	24	nest	102944	43.31	NA	908.1	0.0040650407
V27	27	tree	NA	NA	SP	0	0
V28	28	tree	NA	NA	SP	0	0
V32	32	tree	NA	NA	SP	0	0
V34	34	tree	NA	NA	SP	0	0
V37	37	tree	NA	NA	SP	0	0
V38	38	tree	NA	NA	SP	0	0
V39	39	tree	NA	NA	SP	0	0
V40	40	tree	NA	NA	SP	0	0
V42	42	tree	NA	NA	sycamore	0	0
V45	45	tree	NA	NA	silver birch	0	0
V46	46	tree	NA	NA	SP	0	0
V48	48	tree	NA	NA	SP	0	0
V49	49	tree	NA	NA	SP	0	0
V50	50	tree	NA	NA	SP	0	0
V53	53	tree	NA	NA	sycamore	0	0
V56	56	tree	NA	NA	SP	0	0
V57	57	tree	NA	NA	SP	0	0
V60	60	tree	NA	NA	SP	0	0
V61	61	tree	NA	NA	SP	0	0
V62	62	tree	NA	NA	SP	0	0
V64	64	tree	NA	NA	sycamore	0	0
V65	65	tree	NA	NA	SP	0	0

	node.id	type	sizes	canopy	treespecies	totalforageweight	norm.between
V3	3	nest	8096	33.74	NA	95	0.0852130326
V5	5	nest	18086	14.62	NA	0	0
V6	6	nest	64745	34.28	NA	3282.15	0.4611528822
V8	8	nest	1189	50.75	NA	0	0.4912280702
V9	9	nest	136792	46.44	NA	2735.26	0.7794486216
V15	15	nest	35030	54.2	NA	417.83	0.1127819549
V16	16	nest	67554	18.75	NA	11380	0.0852130326
V17	17	nest	77525	50.26	NA	7152	0.8771929825
V18	18	nest	67127	21.02	NA	5688.36	0.2481203008
V20	20	nest	331313	54.56	NA	56743.25	1
V24	24	nest	285138	47.35	NA	9562.35	0.015037594
V27	27	tree	NA	NA	sycamore	0	0
V28	28	tree	NA	NA	silver birch	0	0
V32	32	tree	NA	NA	SP	0	0
V33	33	tree	NA	NA	SP	0	0
V34	34	tree	NA	NA	SP	0	0
V37	37	tree	NA	NA	sycamore	0	0
V38	38	tree	NA	NA	SP	0	0
V39	39	tree	NA	NA	SP	0	0
V40	40	tree	NA	NA	SP	0	0
V42	42	tree	NA	NA	SP	0	0
V45	45	tree	NA	NA	SP	0	0
V46	46	tree	NA	NA	sycamore	0	0
V49	49	tree	NA	NA	SP	0	0
V53	53	tree	NA	NA	SP	0	0
V56	56	tree	NA	NA	SP	0	0
V57	57	tree	NA	NA	SP	0	0
V58	58	tree	NA	NA	SP	0	0
V60	60	tree	NA	NA	SP	0	0
V61	61	tree	NA	NA	SP	0	0
V64	64	tree	NA	NA	sycamore	0	0
V65	65	tree	NA	NA	SP	0	0
V66	66	tree	NA	NA	SP	0	0
V67	67	tree	NA	NA	SP	0	0
V68	68	tree	NA	NA	SP	0	0
V69	69	tree	NA	NA	SP	0	0
V70	70	tree	NA	NA	SP	0	0
V71	71	nest	3369	41.48	NA	1	0
V72	72	nest	8283	49.36	NA	0	0
V73	73	nest	5491	50.21	NA	58.93	0.7669172932
V74	74	tree	NA	NA	SP	0	0

	node.id	type	sizes	canopy	treespecies	totalforageweight	norm.between
V1	1	nest	6998	0	NA	0	0.3333333333
V2	2	nest	87976	0	NA	0	0
V3	3	nest	4038	37.17	NA	8090	1
V4	4	nest	21422	35.99	NA	1931.4	0.6666666667
V6	6	tree	NA	NA	oak	0	0
V7	7	tree	NA	NA	oak	0	0

	node.id	type	sizes	canopy	treespecies	totalforageweight	norm.between
V2	2	nest	93883	0	NA	1393	0
V3	3	nest	290	6.17	NA	1351.03	0.6
V4	4	nest	4041	35.41	NA	1450	1
V6	6	tree	NA	NA	oak	0	0
V7	7	tree	NA	NA	oak	0	0

	node.id	type	sizes	canopy	treespecies	totalforageweight	norm.between
V2	2	nest	83143	0.04	NA	0	1
V3	3	nest	13271	25.24	NA	758.51	1
V4	4	nest	26988	41.14	NA	580	0.5
V5	5	nest	21113	0.25	NA	0	0
V6	6	tree	NA	NA	oak	0	0
V7	7	tree	NA	NA	oak	0	0

	node.id	type	sizes	canopy	treespecies	totalforageweight	norm.between
V2	2	nest	35117	0	NA	4638.69	1
V3	3	nest	7620	17.98	NA	1351.03	0
V4	4	nest	9955	49.83	NA	1450	0.5
V5	5	nest	3414	0	NA	0	0
V6	6	tree	NA	NA	oak	0	0
V7	7	tree	NA	NA	oak	0	0

	node.id	type	sizes	canopy	treespecies	totalforageweight	norm.between
V2	2	nest	47275	NA	NA	933.31	1
V3	3	nest	2284	34.21	NA	2022.5	1
V4	4	nest	18931	52.95	NA	725	1
V5	5	nest	875	0	NA	0	0
V6	6	tree	NA	NA	oak	0	0
V7	7	tree	NA	NA	oak	0	0

	node.id	type	sizes	canopy	treespecies	totalforageweight	norm.between
V1	1	nest	39297	0	NA	1258.95	0.4375
V2	2	nest	53492	19.76	NA	1501.83	1
V3	3	nest	6006	0	NA	0	0.75
V4	4	nest	13837	0	NA	0	0.9375
V5	5	nest	37035	0	NA	3570	0.75
V6	6	nest	15784	42.69	NA	307	0.4375
V10	10	tree	NA	NA	oak	0	0
V11	11	tree	NA	NA	oak	0	0
V12	12	tree	NA	NA	oak	0	0

IIb.2013a

	node.id	type	sizes	canopy	treespecies	totalforageweight	norm.between
V1	1	nest	51823	1.59	NA	1160.85	0.4117647059
V2	2	nest	8806	8.26	NA	1057.47	1
V4	4	nest	7081	0.05	NA	0	1
V5	5	nest	62641	6.43	NA	3327.14	0.0588235294
V6	6	nest	36329	10.63	NA	614	0
V7	7	nest	11758	0	NA	0	0
V10	10	tree	NA	NA	oak	0	0
V11	11	tree	NA	NA	oak	0	0
V12	12	tree	NA	NA	oak	0	0

	node.id	type	sizes	canopy	treespecies	totalforageweight	norm.between
V1	1	nest	20350	0	NA	1821.7	0.3043478261
V5	5	nest	77892	10.88	NA	1480.48	1
V6	6	nest	10583	22.8	NA	0	0
V7	7	nest	2017	0	NA	0	0.3043478261
V8	8	nest	83143	0.35	NA	0	0
V9	9	nest	21113	46.96	NA	527.05	0
V10	10	tree	NA	NA	oak	0	0
V11	11	tree	NA	NA	oak	0	0
V12	12	tree	NA	NA	oak	0	0

IIb.2014a

	node.id	type	sizes	canopy	treespecies	totalforageweight	norm.between
V1	1	nest	9380	0	NA	2730.45	0.65625
V5	5	nest	48856	4.39	NA	1428	0.65625
V6	6	nest	2841	9.9	NA	0	0.53125
V7	7	nest	4939	0.02	NA	0	0
V9	9	nest	1560	44.8	NA	2219.55	0.8125
V10	10	tree	NA	NA	oak	0	0
V11	11	tree	NA	NA	oak	0	0
V12	12	tree	NA	NA	oak	0	0
V13	13	tree	NA	NA	oak	0	0
V14	14	nest	5265	0	NA	0	0
V15	15	nest	1938	0	NA	0	0.84375
V16	16	nest	17795	19.05	NA	532.8	1
V17	17	nest	2015	9.81	NA	0	0.375

	node.id	type	sizes	canopy	treespecies	totalforageweight	norm.between
V1	1	nest	5649	0	NA	0	0.6444444444
V5	5	nest	22630	11.19	NA	2377.62	0.4222222222
V6	6	nest	1689	21.85	NA	0	0
V7	7	nest	17465	0	NA	0	0
V9	9	nest	3330	46.5	NA	1877.85	0.2444444444
V10	10	tree	NA	NA	oak	0	0
V11	11	tree	NA	NA	oak	0	0
V12	12	tree	NA	NA	oak	0	0
V13	13	tree	NA	NA	oak	0	0
V14	14	nest	3211	0	NA	0	0
V15	15	nest	4773	8.68	NA	0	0.7111111111
V16	16	nest	28828	26.83	NA	2863.8	1
V18	18	nest	1991	6.71	NA	1854	0.2444444444

	node.id	type	sizes	canopy	treespecies	totalforageweight	norm.between
V1	1	nest	14796	41.71	NA	0	0
V2	2	nest	98410	48.81	NA	5100	0.4509803922
V3	3	nest	1827	63.42	NA	0	0.5882352941
V4	4	nest	6967	37.86	NA	0	0
V5	5	nest	27142	48.43	NA	289.71	1
V6	6	nest	114179	50.48	NA	2000	0.9215686275
V7	7	nest	15072	37.61	NA	0	0.7058823529
V8	8	nest	44791	21.74	NA	2913.75	0.6274509804
V9	9	nest	20325	32.87	NA	0	0.2352941176
V10	10	nest	41138	12.68	NA	0	0
V15	15	nest	57596	46.63	NA	3600	0
V16	16	nest	68502	33.53	NA	5975	0.0392156863
V17	17	tree	NA	NA	SP	0	0
V18	18	tree	NA	NA	SP	0	0
V19	19	tree	NA	NA	silver birch	0	0
V20	20	tree	NA	NA	SP	0	0
V21	21	tree	NA	NA	SP	0	0
V22	22	tree	NA	NA	SP	0	0

	node.id	type	sizes	canopy	treespecies	totalforageweight	norm.between
V1	1	nest	5402	NA	NA	173.25	0.2112676056
V2	2	nest	21479	NA	NA	629.2	0
V3	3	nest	763	NA	NA	0	0.5492957746
V5	5	nest	38384	NA	NA	87	1
V6	6	nest	41343	NA	NA	2000	0.3943661972
V7	7	nest	42254	NA	NA	218.5	0.7746478873
V11	11	nest	15072	NA	NA	0	1
V12	12	nest	2391	NA	NA	0	0
V13	13	nest	24702	NA	NA	0	0
V14	14	nest	3211	NA	NA	0	0
V15	15	nest	51979	NA	NA	827.6	0.7464788732
V16	16	nest	27620	NA	NA	2600	0.2112676056
V18	18	tree	NA	NA	SP	0	0
V19	19	tree	NA	NA	SP	0	0
V20	20	tree	NA	NA	SP	0	0
V21	21	tree	NA	NA	SP	0	0
V22	22	tree	NA	NA	SP	0	0

	node.id	type	sizes	canopy	treespecies	totalforageweight	norm.between
V2	2	nest	38352	46.63	NA	61.6	0
V5	5	nest	69855	41.79	NA	870	0.9428571429
V6	6	nest	39464	47.24	NA	666	0.6
V7	7	nest	56595	19.52	NA	918	1
V11	11	nest	34756	45.75	NA	131	0.3142857143
V12	12	nest	3984	23.3	NA	0	0
V15	15	nest	90551	9.37	NA	1832.4	0.8285714286
V16	16	nest	118171	35.4	NA	1300	0.3142857143
V18	18	tree	NA	NA	SP	0	0
V19	19	tree	NA	NA	SP	0	0
V20	20	tree	NA	NA	SP	0	0
V21	21	tree	NA	NA	SP	0	0
V22	22	tree	NA	NA	SP	0	0

	node.id	type	sizes	canopy	treespecies	totalforageweight	norm.between
V2	2	nest	15389	48.9	NA	0	0
V5	5	nest	5869	41.69	NA	58.29	0.387755102
V6	6	nest	31734	47.39	NA	2000	0.4285714286
V7	7	nest	51374	29.14	NA	4590	1
V11	11	nest	21334	26.11	NA	87.77	0.5918367347
V15	15	nest	59990	33.31	NA	2598.8	1
V16	16	nest	30286	45.79	NA	865.8	0.6734693878
V18	18	tree	NA	NA	SP	0	0
V19	19	tree	NA	NA	SP	0	0
V20	20	tree	NA	NA	SP	0	0
V21	21	tree	NA	NA	SP	0	0
V22	22	tree	NA	NA	SP	0	0
V23	23	tree	NA	NA	SP	0	0
V24	24	nest	8931	0	NA	0	0
V25	25	nest	71110	16.65	NA	2920.08	0.2653061224

	node.id	type	sizes	canopy	treespecies	totalforageweight	norm.between
V2	2	nest	58506	19.69	NA	176	0
V5	5	nest	24890	36.69	NA	87	0.7703703704
V6	6	nest	39697	26.56	NA	2000	0.3037037037
V7	7	nest	28490	26.35	NA	2295	0.8888888889
V11	11	nest	45318	NA	NA	0	0.4222222222
V15	15	nest	134005	28.82	NA	3998.8	1
V16	16	nest	166815	32.64	NA	1300	0.6296296296
V18	18	tree	NA	NA	SP	0	0
V19	19	tree	NA	22.64	SP	0	0
V20	20	tree	NA	NA	SP	0	0
V21	21	tree	NA	NA	SP	0	0
V22	22	tree	NA	NA	SP	0	0
V23	23	tree	NA	NA	SP	0	0
V24	24	nest	63484	0	NA	0	0.2962962963
V25	25	nest	65064	15.66	NA	7051	0.3037037037
V26	26	nest	156	40.67	NA	47	0.1555555556
V27	27	nest	737	16.07	NA	0	0.1555555556
V28	28	nest	13915	0	NA	0	0
V29	29	nest	5869	14.64	NA	443.22	0
V30	30	nest	6199	31.93	NA	960	0.2962962963
V31	31	nest	338	21.88	NA	395	0
V32	32	nest	1474	19.17	NA	530	0.1333333333
V33	33	tree	NA	NA	SP	0	0

	node.id	type	sizes	canopy	treespecies	totalforage	weight	norm.between
V1	1	nest	4010	11.76	NA		0	0
V2	2	nest	43874	34.67	NA		0	0.34905660377358
V3	3	nest	6832	19.97	NA		0	0.19811320754717
V4	4	tree	NA	NA	SP		0	0
V5	5	nest	53863	48.83	NA		1961.79	0.60377358490566
V6	6	nest	28828	52.05	NA		1692.5	0.43396226415094
V7	7	nest	851	50.53	NA		0	0.19811320754717
V8	8	nest	185500	19.85	NA		2040	1
V9	9	nest	24694	21.4	NA		1506	0.58490566037736
V10	10	nest	36565	0.29	NA		2262.25	0.17924528301887
V11	11	nest	83482	10.04	NA		1475	0.33018867924528
V12	12	nest	27609	0.04	NA		0	0
V13	13	tree	NA	NA	SP		0	0
V14	14	tree	NA	NA	SP		0	0
V16	16	tree	NA	NA	SP		0	0
V17	17	tree	NA	NA	SP		0	0
V18	18	tree	NA	NA	SP		0	0
V19	19	tree	NA	NA	SP		0	0
V20	20	tree	NA	NA	SP		0	0
V21	21	tree	NA	NA	SP		0	0
V23	23	nest	47844	39.46	NA		0	0.59433962264151

	node.id	type	sizes	canopy	treespecies	totalforage	weight	norm.between
V2	2	nest	1808	24.55	NA		642.5	0.21794871794872
V3	3	nest	23678	35.75	NA		292.25	0.34615384615385
V5	5	nest	10356	48.56	NA		160	0.56410256410256
V7	7	nest	16746	54.57	NA		543.21	1
V8	8	nest	15210	15.28	NA		0	0.7051282051282
V9	9	nest	3397	31.36	NA		179.19	0.88461538461539
V10	10	nest	15210	5.16	NA		0	0.61538461538461
V11	11	nest	57005	13.13	NA		3100	0.53846153846154
V12	12	tree	NA	NA	SP		0	0
V14	14	tree	NA	NA	SP		0	0
V16	16	tree	NA	NA	SP		0	0
V17	17	tree	NA	NA	SP		0	0
V19	19	tree	NA	NA	SP		0	0
V20	20	tree	NA	NA	SP		0	0
V21	21	tree	NA	NA	SP		0	0
V22	22	tree	NA	NA	SP		0	0
V23	23	nest	42934	37.95	NA		0	0

	node.id	type	sizes	canopy	treespecies	totalforageweight	norm.between
V2	2	nest	2354	40.21	NA	1837.55	0.29268292682927
V3	3	nest	75775	44.09	NA	1250.55	1
V5	5	nest	18874	38.47	NA	842.49	0.29268292682927
V7	7	nest	16945	52.32	NA	2713.95	0.75609756097561
V8	8	nest	117237	10.95	NA	1644.95	0.78048780487805
V11	11	nest	66660	1.26	NA	936.65	0.29268292682927
V12	12	tree	NA	NA	SP	0	0
V13	13	tree	NA	NA	SP	0	0
V14	14	tree	NA	NA	SP	0	0
V16	16	tree	NA	NA	SP	0	0
V19	19	tree	NA	NA	SP	0	0
V20	20	tree	NA	NA	SP	0	0
V21	21	tree	NA	NA	SP	0	0
V22	22	tree	NA	NA	SP	0	0

	node.id	type	sizes	canopy	treespecies	totalforageweight	norm.between
V3	3	nest	125130	39.02	NA	350	0
V5	5	nest	5538	30.44	NA	413	0.53846153846154
V7	7	nest	26257	37.47	NA	619.69	1
V8	8	nest	42370	17.3	NA	0	0.84615384615385
V11	11	nest	44058	3.76	NA	989.45	0.57692307692308
V13	13	tree	NA	NA	SP	0	0
V14	14	tree	NA	NA	SP	0	0
V16	16	tree	NA	NA	SP	0	0
V20	20	tree	NA	NA	SP	0	0
V21	21	tree	NA	NA	SP	0	0
V22	22	tree	NA	NA	SP	0	0
V24	24	nest	6639	0.01	NA	0	0.84615384615385
V25	25	nest	2104	0.01	NA	1021.52	0.96153846153846

	node.id	type	sizes	canopy	treespecies	totalforageweight	norm.between
V3	3	nest	83560	43.08	NA	3454.75	1
V5	5	nest	3319	45.81	NA	126.5	0.58823529411765
V7	7	nest	35407	33.38	NA	1059.97	0.23529411764706
V8	8	nest	83343	13.04	NA	3778.95	1
V11	11	nest	44505	4.82	NA	2181.15	0.3921568627451
V12	12	tree	NA	NA	SP	0	0
V14	14	tree	NA	NA	SP	0	0
V16	16	tree	NA	NA	SP	0	0
V17	17	tree	NA	NA	SP	0	0
V20	20	tree	NA	NA	SP	0	0
V21	21	tree	NA	NA	SP	0	0
V22	22	tree	NA	NA	SP	0	0
V24	24	nest	41970	NA	NA	0	0
V25	25	nest	6089	0	NA	1269.2	0.03921568627451

	node.id	type	sizes	canopy	treespecie	totalforag	norm.between
V1	1	nest	522	0	NA	0	0
V2	2	nest	11796	0	NA	0	0.173913
V3	3	nest	11422	0	NA	0	0
V4	4	nest	265005	0	NA	0	0.478261
V6	6	nest	29562	32.47	NA	1762.5	0.565217
V7	7	nest	149033	18.24	NA	5050	1
V8	8	nest	8771	5.06	NA	0	0
V9	9	nest	92640	0.16	NA	0	0
V10	10	nest	20773	0.09	NA	0	0
V11	11	nest	6731	27.47	NA	0	0
V12	12	nest	52877	31.85	NA	0	0.826087
V13	13	nest	74212	28.23	NA	831.95	0.728261
V14	14	nest	19130	24.06	NA	0	0.326087
V15	15	nest	1523	29.89	NA	68	0.173913
V23	23	tree	NA	NA	SP	0	0
V24	24	tree	NA	NA	SP	0	0
V25	25	tree	NA	NA	larch	0	0
V26	26	tree	NA	NA	SP	0	0

	node.id	type	sizes	canopy	treespecie	totalforag	norm.between
V2	2	nest	2494	0	NA	0	0
V4	4	nest	39464	0.03	NA	0	0.389831
V6	6	nest	2405	35.06	NA	7050	0.508475
V7	7	nest	5101	10.35	NA	0	0
V10	10	nest	6598	5.64	NA	0	0.372881
V12	12	nest	33628	24.21	NA	2797.2	1
V16	16	nest	25843	1.73	NA	292.5	0.20339
V17	17	nest	20857	17.38	NA	0	0.508475
V18	18	nest	10136	37.55	NA	313.78	0.389831
V19	19	nest	4725	32.47	NA	0	0
V23	23	tree	NA	NA	SP	0	0
V24	24	tree	NA	NA	SP	0	0
V26	26	tree	NA	7.87	SP	0	0
V27	27	nest	8308	NA	NA	0	0

	node.id	type	sizes	canopy	treespecie	totalforag	norm.between
V2	2	nest	1342	0	NA	0	0
V4	4	nest	56393	0	NA	1350	0.294118
V6	6	nest	306	56.45	NA	232.65	0
V7	7	nest	57714	12.1	NA	1010	0.882353
V10	10	nest	48985	15.99	NA	67	1
V11	11	tree	NA	NA	NA	0	0
V12	12	nest	8931	43.95	NA	277.2	0
V16	16	nest	34934	10.65	NA	0	0.764706
V20	20	nest	369	0.01	NA	0	0
V21	21	nest	369	34.34	NA	0	0
V22	22	nest	6254	14.38	NA	0	0.294118
V23	23	tree	NA	NA	larch	0	0

	node.id	type	sizes	canopy	treespecie	totalforag	norm.between
V4	4	nest	1527	1.66	NA	0	0
V6	6	nest	4466	37.24	NA	0	0.291667
V7	7	nest	14457	0.86	NA	0	0
V10	10	nest	2187	8.53	NA	0	0.291667
V12	12	nest	34683	26.78	NA	4200	1
V16	16	nest	13218	3.37	NA	0	0
V22	22	nest	16070	33.06	NA	0	0
V23	23	tree	NA	NA	larch	0	0
V28	28	nest	15177	NA	NA	0	0.5

	node.id	type	sizes	canopy	treespecie	totalforag	norm.between
V7	7	nest	9597	50.74	NA	0	0
V12	12	nest	9599	0	NA	840	1
V23	23	tree	NA	NA	larch	0	0

	node.id	type	sizes	canopy	treespecie	totalforag	norm.between
V1	1	nest	6750	4.14	NA	506.01	0
V2	2	nest	6750	0	NA	0	0
V3	3	nest	10117	5.46	NA	1941.39	0.381579
V4	4	nest	19247	14.44	NA	10755	1
V5	5	nest	21237	3.31	NA	0	0
V6	6	nest	9711	6.74	NA	0	0.631579
V7	7	nest	110039	64	NA	0	0.598684
V8	8	nest	5019	53.36	NA	1410.7	0.269737
V9	9	nest	14055	68.38	NA	2297.7	0.138158
V10	10	nest	48228	36.98	NA	1910	0.138158
V11	11	nest	19163	34.57	NA	0	0
V12	12	nest	37349	42.4	NA	5118.86	0.559211
V13	13	nest	119	42.93	NA	41	0.098684
V14	14	nest	47703	40.89	NA	1884.96	0.269737
V16	16	tree	NA	NA	silver birch	0	0
V17	17	tree	NA	NA	silver birch	0	0
V18	18	tree	NA	NA	oak	0	0
V19	19	tree	NA	NA	silver birch	0	0
V20	20	tree	NA	NA	larch	0	0
V21	21	tree	NA	NA	silver birch	0	0
V22	22	tree	NA	NA	silver birch	0	0
V23	23	tree	NA	NA	silver birch	0	0
V24	24	tree	NA	NA	silver birch	0	0

	node.id	type	sizes	canopy	treespecie	totalforag	norm.between
V1	1	nest	169	5.35	NA	0	0
V2	2	nest	4500	2.86	NA	215	0.184466
V3	3	nest	3617	0.11	NA	262.35	0.495146
V4	4	nest	2115	3.57	NA	0	0.941748
V5	5	nest	9969	37.61	NA	516	0.466019
V6	6	nest	791	6.72	NA	0	0.699029
V7	7	nest	75395	26.39	NA	4195.75	0.359223
V9	9	nest	10862	56.68	NA	462.3	0.23301
V10	10	nest	10303	2.8	NA	3820	1
V11	11	nest	6465	16.03	NA	0	0.815534
V12	12	nest	15078	30.04	NA	20913.6	0.805825
V14	14	nest	8540	18.68	NA	1140.37	0
V16	16	tree	NA	NA	silver birc	0	0
V17	17	tree	NA	NA	silver birc	0	0
V18	18	tree	NA	NA	oak	0	0
V19	19	tree	NA	NA	silver birc	0	0
V20	20	tree	NA	NA	larch	0	0
V21	21	tree	NA	NA	silver birc	0	0
V22	22	tree	NA	NA	silver birc	0	0
V23	23	tree	NA	NA	silver birc	0	0
V24	24	tree	NA	NA	silver birc	0	0

	node.id	type	sizes	canopy	treespecie	totalforag	norm.between
V1	1	nest	7869	32.8	NA	0	0
V2	2	nest	71700	0	NA	359.05	0.333333
V3	3	nest	21821	3.87	NA	0	0.454545
V4	4	nest	47309	9.17	NA	0	0.565657
V5	5	nest	54942	22.1	NA	340.56	0.656566
V6	6	nest	29106	28.31	NA	0	0
V7	7	nest	64475	43.18	NA	0	0.111111
V9	9	nest	26396	58.42	NA	690	0.666667
V10	10	nest	65653	21.95	NA	637.94	0
V12	12	nest	42327	36.34	NA	8585.25	0.858586
V14	14	nest	42327	46	NA	200.86	0.171717
V15	15	nest	37054	48.95	NA	5042.5	0.414141
V16	16	tree	NA	NA	larch	0	0
V17	17	tree	NA	NA	silver birc	0	0
V18	18	tree	NA	NA	silver birc	0	0
V20	20	tree	NA	NA	silver birc	0	0
V21	21	tree	NA	NA	oak	0	0
V22	22	tree	NA	NA	silver birc	0	0
V24	24	tree	NA	NA	silver birc	0	0

	node.id	type	sizes	canopy	treespecie	totalforag	norm.between
V1	1	nest	1628	21.4	NA	506.01	0
V2	2	nest	5247	11.41	NA	215	0.27907
V3	3	nest	2271	4.54	NA	0	0.395349
V4	4	nest	11553	2.19	NA	0	0.496124
V5	5	nest	17790	38.96	NA	2064	1
V6	6	nest	36971	34.65	NA	1298.7	0.108527
V7	7	nest	60651	50.24	NA	1411.15	0.395349
V9	9	nest	14570	60.78	NA	2297.7	0
V10	10	nest	3475	39.67	NA	0	0.03876
V12	12	nest	75136	22.5	NA	8263.86	0.51938
V14	14	nest	4814	32.73	NA	1014.8	0.147287
V15	15	nest	13812	37.52	NA	695.24	0.286822
V16	16	tree	NA	NA	oak	0	0
V17	17	tree	NA	NA	silver birc	0	0
V18	18	tree	NA	NA	larch	0	0
V19	19	tree	NA	NA	silver birc	0	0
V20	20	tree	NA	NA	silver birc	0	0
V21	21	tree	NA	NA	silver birc	0	0
V22	22	tree	NA	NA	silver birc	0	0
V24	24	tree	NA	NA	oak	0	0
V25	25	nest	15286	27.84	NA	1814.85	0.744186

	node.id	type	sizes	canopy	treespecie	totalforag	norm.between
V1	1	nest	1311	23	NA	203.01	0.170213
V3	3	nest	1639	0	NA	0	0.319149
V4	4	nest	10080	0	NA	0	0.446809
V5	5	nest	9003	38.81	NA	3436.56	1
V6	6	nest	11681	25.52	NA	0	0.680851
V7	7	nest	4478	51.86	NA	0	0.212766
V9	9	nest	24513	62.42	NA	407.1	0
V10	10	nest	41934	17.47	NA	3820	0.319149
V12	12	nest	44233	33.63	NA	23539.85	0.617021
V15	15	nest	24919	33.65	NA	0	0
V16	16	tree	NA	NA	larch	0	0
V17	17	tree	NA	NA	silver birc	0	0
V18	18	tree	NA	NA	silver birc	0	0
V20	20	tree	NA	NA	silver birc	0	0
V21	21	tree	NA	NA	oak	0	0
V22	22	tree	NA	NA	silver birc	0	0
V24	24	tree	NA	NA	silver birc	0	0
V26	26	nest	1066	43.69	NA	726	0

	node.id	type	sizes	canopy	treespeci	totalforag	norm.between
V1	1	nest	35551	0.22	NA	963	0.543478
V2	2	nest	13633	0.03	NA	787.25	0.195652
V3	3	nest	7532	0	NA	0	0
V4	4	nest	34221	0	NA	5670	0.23913
V5	5	nest	45083	0	NA	3829.5	1
V6	6	nest	28213	8.15	NA	16270.8	0.652174
V7	7	nest	70952	0	NA	3225	0.391304
V8	8	tree	NA	NA	SP	0	0
V9	9	tree	NA	NA	oak	0	0
V10	10	tree	NA	NA	oak	0	0
V11	11	tree	NA	NA	oak	0	0
V12	12	tree	NA	NA	oak	0	0
V13	13	tree	NA	NA	oak	0	0

	node.id	type	sizes	canopy	treespecie	totalforag	norm.between
V1	1	nest	18391	0	NA	963	0
V2	2	nest	285	0	NA	340.75	0.282051
V3	3	nest	1080	0	NA	0	0.74359
V4	4	nest	27103	0.02	NA	5670	1
V5	5	nest	21760	0	NA	5750	0.820513
V6	6	nest	8816	14.97	NA	17870	0.769231
V7	7	nest	114238	1.71	NA	7754.25	0.923077
V8	8	tree	NA	NA	SP	0	0
V9	9	tree	NA	NA	oak	0	0
V10	10	tree	NA	NA	oak	0	0
V11	11	tree	NA	NA	oak	0	0
V12	12	tree	NA	NA	oak	0	0
V13	13	tree	NA	NA	oak	0	0

	node.id	type	sizes	canopy	treespecie	totalforag	norm.between
V2	2	nest	17724	1.3	NA	387.75	0
V5	5	nest	38296	10.45	NA	1437.5	0.6
V6	6	nest	14721	30.56	NA	6955	1
V7	7	nest	101914	5.16	NA	564	0
V8	8	tree	NA	NA	oak	0	0
V9	9	tree	NA	NA	oak	0	0
V10	10	tree	NA	NA	oak	0	0
V11	11	tree	NA	NA	oak	0	0
V12	12	tree	NA	NA	SP	0	0

	node.id	type	sizes	canopy	treespecie	totalforag	norm.between
V2	2	nest	9857	1.36	NA	1962.25	0.257143
V5	5	nest	4591	0.38	NA	7902.5	1
V6	6	nest	8858	17.61	NA	7120	0.485714
V7	7	nest	15012	0.81	NA	3225	0
V8	8	tree	NA	NA	SP	0	0
V9	9	tree	NA	NA	oak	0	0
V10	10	tree	NA	NA	oak	0	0
V11	11	tree	NA	NA	oak	0	0
V12	12	tree	NA	NA	oak	0	0
V13	13	tree	NA	NA	oak	0	0
V14	14	nest	1619	0.18	NA	1387.5	0.657143

	node.id	type	sizes	canopy	treespecie	totalforag	norm.between
V2	2	nest	17044	0.08	NA	1962.25	0
V5	5	nest	19144	3.18	NA	6542.5	1
V6	6	nest	27752	20.61	NA	12849.9	0.472222
V7	7	nest	8401	2.12	NA	535.35	0
V8	8	tree	NA	NA	oak	0	0
V9	9	tree	NA	NA	oak	0	0
V10	10	tree	NA	NA	oak	0	0
V11	11	tree	NA	NA	oak	0	0
V12	12	tree	NA	NA	SP	0	0
V15	15	nest	20453	4.58	NA	1275	0
V16	16	nest	2659	2.61	NA	524	0.25
V17	17	nest	5115	2.77	NA	0	0
V18	18	nest	2579	13.89	NA	224.75	0

	node.id	type	sizes	canopy	treespecie	totalforag	norm.between
V1	1	nest	7413	43.24	NA	176	0.23913
V2	2	nest	41890	56.1	NA	365.2	0.804348
V3	3	nest	54744	43.97	NA	609.35	1
V4	4	nest	582	44.92	NA	0	0
V5	5	nest	210	30.87	NA	0	0
V6	6	nest	247416	49.47	NA	21272.05	0.652174
V7	7	tree	NA	NA	larch	0	0
V8	8	tree	NA	NA	SP	0	0
V9	9	tree	NA	NA	larch	0	0
V10	10	tree	NA	NA	larch	0	0
V12	12	tree	NA	NA	larch	0	0
V13	13	tree	NA	NA	SP	0	0
V14	14	tree	NA	NA	larch	0	0

	node.id	type	sizes	canopy	treespecie	totalforag	norm.between
V3	3	nest	19262	40.3	NA	1435.35	0.566667
V6	6	nest	91502	18.77	NA	5518.62	1
V7	7	tree	NA	NA	larch	0	0
V8	8	tree	NA	NA	larch	0	0
V9	9	tree	NA	NA	SP	0	0
V10	10	tree	NA	NA	larch	0	0
V11	11	nest	11983	38.22	NA	3177.6	0.966667
V12	12	tree	NA	NA	SP	0	0
V13	13	tree	NA	NA	larch	0	0
V14	14	tree	NA	NA	SP	0	0
V15	15	tree	NA	NA	larch	0	0

	node.id	type	sizes	canopy	treespecie	totalforag	norm.between
V1	1	nest	6750	42.42	NA	294.8	0.310345
V3	3	nest	23471	62.34	NA	530	0.551724
V6	6	nest	227526	51.51	NA	9265.82	0.827586
V7	7	tree	NA	NA	larch	0	0
V8	8	tree	NA	NA	SP	0	0
V10	10	tree	NA	NA	SP	0	0
V11	11	nest	22821	44.37	NA	1325.62	1
V12	12	tree	NA	NA	SP	0	0
V13	13	tree	NA	NA	larch	0	0
V14	14	tree	NA	NA	larch	0	0
V15	15	tree	NA	NA	SP	0	0

	node.id	type	sizes	canopy	treespecie	totalforag	norm.between
V1	1	nest	1342	24.44	NA	220	0.184211
V3	3	nest	21479	46.52	NA	545	0.5
V6	6	nest	93466	38.53	NA	21211.61	1
V7	7	tree	NA	NA	larch	0	0
V8	8	tree	NA	NA	SP	0	0
V9	9	tree	NA	NA	SP	0	0
V10	10	tree	NA	NA	SP	0	0
V11	11	nest	13275	36.06	NA	0	0.736842
V12	12	tree	NA	NA	larch	0	0
V13	13	tree	NA	NA	larch	0	0
V14	14	tree	NA	NA	SP	0	0
V16	16	nest	819	NA	NA	820	0.184211
V17	17	tree	NA	NA	larch	0	0
V18	18	tree	NA	NA	SP	0	0
V19	19	tree	NA	NA	SP	0	0
V20	20	nest	14784	37.93	NA	376.75	0.828947

	node.id	type	sizes	canopy	treespecie	totalforag	norm.between
V3	3	nest	668	40.9	NA	2136	0.403509
V6	6	nest	384166	28.09	NA	36435.66	1
V7	7	tree	NA	NA	larch	0	0
V8	8	tree	NA	NA	larch	0	0
V9	9	tree	NA	NA	SP	0	0
V10	10	tree	NA	NA	SP	0	0
V11	11	nest	23626	30.39	NA	2560	0
V12	12	tree	NA	NA	larch	0	0
V13	13	tree	NA	NA	larch	0	0
V14	14	tree	NA	NA	SP	0	0
V15	15	tree	NA	NA	larch	0	0
V17	17	tree	NA	NA	larch	0	0
V18	18	tree	NA	NA	larch	0	0
V20	20	nest	2217	32.76	NA	877.36	0.929825

	node.id	type	sizes	canopy	treespecies	totalforageweight	norm.between
V1	1	nest	26225	30.37	NA	1950	0
V2	2	nest	4591	20.9	NA	2250	0
V3	3	nest	25124	22.98	NA	2650	0.43269230769231
V4	4	nest	288380	16.56	NA	13270	1
V5	5	nest	134348	14.48	NA	9213.1	0.16346153846154
V6	6	nest	50269	30.65	NA	5329.15	0.64423076923077
V7	7	nest	158272	2.83	NA	11341.89	0.46153846153846
V8	8	nest	45471	0	NA	0	0.30769230769231
V9	9	nest	32805	0	NA	3120	0.16346153846154
V19	19	tree	NA	NA	sycamore	0	0
V20	20	tree	NA	NA	silver birch	0	0
V21	21	tree	NA	NA	sycamore	0	0
V22	22	tree	NA	NA	silver birch	0	0
V23	23	tree	NA	NA	silver birch	0	0
V24	24	tree	NA	NA	silver birch	0	0
V25	25	tree	NA	NA	silver birch	0	0
V26	26	tree	NA	NA	oak	0	0
V27	27	tree	NA	NA	sycamore	0	0
V28	28	tree	NA	NA	oak	0	0

	node.id	type	sizes	canopy	treespecies	totalforageweight	norm.between
V1	1	nest	2120	18.25	NA	649.35	0.16216216216216
V3	3	nest	712	24.65	NA	2867	0.58558558558559
V4	4	nest	74569	18.67	NA	5275	1
V5	5	nest	17017	20.04	NA	7763.1	0.81981981981982
V6	6	nest	8116	23.76	NA	1525	0.43243243243243
V7	7	nest	8746	6.25	NA	7815.04	0.31531531531531
V8	8	nest	754	0	NA	0	0.30630630630631
V9	9	nest	15708	0	NA	7800	0.16216216216216
V10	10	nest	503	6.23	NA	0	0.74774774774775
V11	11	nest	68	0	NA	0	0
V12	12	nest	1414	0	NA	1050	0.1981981981982
V19	19	tree	NA	NA	sycamore	0	0
V20	20	tree	NA	NA	silver birch	0	0
V21	21	tree	NA	NA	silver birch	0	0
V22	22	tree	NA	NA	silver birch	0	0
V23	23	tree	NA	NA	silver birch	0	0
V24	24	tree	NA	NA	oak	0	0
V26	26	tree	NA	NA	sycamore	0	0
V27	27	tree	NA	NA	silver birch	0	0
V28	28	tree	NA	NA	sycamore	0	0

	node.id	type	sizes	canopy	treespecies	totalforageweight	norm.between
V1	1	nest	39483	44.77	NA	2141.73	0.099173553719008
V3	3	nest	3741	51.15	NA	0	0.090909090909091
V4	4	nest	155069	37.06	NA	10990	1
V5	5	nest	29379	29	NA	5730.1	0.590909090909
V6	6	nest	57450	55.62	NA	810	0.272727272727
V7	7	nest	22314	21.78	NA	1456.69	0.19421487603306
V8	8	nest	17554	0	NA	0	0.34710743801653
V9	9	nest	561	0	NA	0	0.272727272727
V10	10	nest	1993	1.56	NA	0	0.099173553719008
V11	11	nest	858	0	NA	0	0
V12	12	nest	871	56.58	NA	766.4	0.4297520661157
V13	13	nest	735	0	NA	0	0.1900826446281
V14	14	nest	910	0	NA	0	0.099173553719008
V15	15	nest	1294	0	NA	0	0
V16	16	nest	1544	0	NA	0	0.1900826446281
V17	17	nest	910	0	NA	672.56	0.099173553719008
V18	18	nest	550	45.4	NA	0	0.0082644628099174
V19	19	tree	NA	NA	sycamore	0	0
V20	20	tree	NA	NA	silver birch	0	0
V21	21	tree	NA	NA	silver birch	0	0
V22	22	tree	NA	NA	silver birch	0	0
V23	23	tree	NA	NA	silver birch	0	0
V24	24	tree	NA	NA	oak	0	0
V26	26	tree	NA	NA	sycamore	0	0
V27	27	tree	NA	NA	silver birch	0	0
V28	28	tree	NA	NA	sycamore	0	0

	node.id	type	sizes	canopy	treespecies	totalforageweight	norm.between
V1	1	nest	44068	17.99	NA	2840.72	0.32710280373832
V3	3	nest	1080	31.61	NA	234.5	0.4018691588785
V4	4	nest	68712	42.32	NA	9460	1
V5	5	nest	20159	25.75	NA	11750	0.1588785046729
V6	6	nest	47579	27.4	NA	2577.5	0.42056074766355
V7	7	nest	30169	3.97	NA	5311.31	0.30841121495327
V8	8	nest	15784	0	NA	0	0.52336448598131
V9	9	nest	21334	0	NA	5194.8	0.42056074766355
V10	10	nest	11212	14.81	NA	0	0
V14	14	nest	19130	0	NA	0	0
V15	15	nest	5265	0	NA	1444.2	0.1588785046729
V19	19	tree	NA	NA	silver birch	0	0
V20	20	tree	NA	NA	silver birch	0	0
V22	22	tree	NA	NA	oak	0	0
V23	23	tree	NA	NA	sycamore	0	0
V24	24	tree	NA	NA	silver birch	0	0
V26	26	tree	NA	NA	silver birch	0	0
V27	27	tree	NA	NA	silver birch	0	0
V28	28	tree	NA	NA	silver birch	0	0

	node.id	type	sizes	canopy	treespecies	totalforageweight	norm.between
V1	1	nest	18583	10.48	NA	130.65	0.024691358024691
V3	3	nest	37479	19.85	NA	190.9	0
V4	4	nest	118736	30.79	NA	6868.6	1
V5	5	nest	27728	32.75	NA	7450	0.12345679012346
V6	6	nest	37164	29.74	NA	4643.75	0.34567901234568
V7	7	nest	27505	9.21	NA	669.2	0.12345679012346
V8	8	nest	4564	28.59	NA	0	0.33333333333333
V9	9	nest	33260	13.84	NA	1950	0.24074074074074
V10	10	nest	1080	22.97	NA	0	0
V14	14	nest	4882	0.13	NA	0	0.1358024691358
V15	15	nest	5450	0.77	NA	0	0.4320987654321
V19	19	tree	NA	NA	oak	0	0
V20	20	tree	NA	NA	sycamore	0	0
V22	22	tree	NA	NA	sycamore	0	0
V23	23	tree	NA	NA	silver birch	0	0
V26	26	tree	NA	NA	oak	0	0
V27	27	tree	NA	NA	sycamore	0	0
V28	28	tree	NA	NA	silver birch	0	0
V29	29	nest	5138	0	NA	0	0.12345679012346
V30	30	nest	657	2.73	NA	0	0
V31	31	nest	497	20.96	NA	0	0
V32	32	nest	900	35.31	NA	415.8	0.20987654320988

	node.id	type	sizes	canopy	treespecie	totalforag	norm.between
V1	1	nest	10080	3.02	NA	1182.15	0.166667
V2	2	nest	1342	4.12	NA	0	0.315789
V3	3	nest	13064	14.32	NA	0	0.587719
V4	4	nest	11571	6.46	NA	0	0
V5	5	nest	23428	3.39	NA	4128.99	1
V6	6	nest	70586	0.02	NA	5915	0.947368
V7	7	nest	5512	0	NA	0	0.122807
V8	8	nest	4637	0	NA	0	0.070175
V9	9	nest	78256	0	NA	0	0.04386
V10	10	nest	18056	1.6	NA	0	0.201754
V11	11	nest	90064	7.28	NA	2286.2	0.649123
V12	12	nest	73247	44.03	NA	0	0.570175
V13	13	nest	4964	51.79	NA	1995	0.570175
V18	18	tree	NA	NA	larch	0	0
V19	19	tree	NA	NA	larch	0	0
V20	20	tree	NA	NA	larch	0	0
V21	21	tree	NA	NA	SP	0	0
V22	22	tree	NA	NA	larch	0	0
V23	23	tree	NA	NA	SP	0	0
V24	24	tree	NA	NA	SP	0	0
V25	25	tree	NA	NA	larch	0	0

	node.id	type	sizes	canopy	treespecie	totalforag	norm.between
V5	5	nest	17448	40.69	NA	261.87	0.294118
V6	6	nest	32899	0	NA	0	0.764706
V7	7	nest	246	0	NA	0	0
V8	8	nest	3956	0	NA	0	0.529412
V9	9	nest	10625	0	NA	574.2	0.294118
V10	10	nest	3279	0	NA	0	0.705882
V11	11	nest	34307	0.08	NA	285	1
V14	14	nest	2727	47.32	NA	1890	1
V19	19	tree	NA	NA	SP	0	0
V22	22	tree	NA	NA	SP	0	0
V24	24	tree	NA	NA	SP	0	0
V25	25	tree	NA	NA	larch	0	0

	node.id	type	sizes	canopy	treespecie	totalforag	norm.between
V5	5	nest	12600	0	NA	1591.5	0.522727
V6	6	nest	35117	0	NA	0	1
V7	7	nest	5115	0	NA	0	0.954545
V8	8	nest	13218	0	NA	1324	0.954545
V9	9	nest	40735	0	NA	854.92	0
V11	11	nest	18086	0.11	NA	1630.2	0.272727
V14	14	nest	2021	34.46	NA	270.27	0.727273
V15	15	nest	754	0	NA	0	0
V16	16	nest	707	0	NA	0	0.681818
V17	17	nest	50712	52.57	NA	80	0
V19	19	tree	NA	NA	SP	0	0
V20	20	tree	NA	NA	SP	0	0
V22	22	tree	NA	NA	larch	0	0
V25	25	tree	NA	NA	larch	0	0

	node.id	type	sizes	canopy	treespeci	totalforag	norm.between
V5	5	nest	6132	0.11	NA	749.49	0.407407
V6	6	nest	39271	0	NA	0	0.740741
V7	7	nest	10583	0	NA	0	0.444444
V8	8	nest	4364	0	NA	0	0.62963
V9	9	nest	13500	0	NA	510.4	0.962963
V11	11	nest	23238	0.85	NA	1467.8	1
V14	14	nest	12207	49.03	NA	0	0
V17	17	nest	5915	57.4	NA	400	0.740741
V19	19	tree	NA	NA	larch	0	0
V22	22	tree	NA	NA	SP	0	0
V23	23	tree	NA	NA	larch	0	0
V25	25	tree	NA	NA	larch	0	0
V26	26	nest	7837	0	NA	0	0

	node.id	type	sizes	canopy	treespeci	totalforag	norm.between
V5	5	nest	9386	0	NA	1002.33	0.285714
V6	6	nest	4069	0	NA	140	0.742857
V7	7	nest	754	0	NA	0	0.8
V8	8	nest	4637	0	NA	1324	1
V9	9	nest	11667	0	NA	854.92	0.285714
V11	11	nest	27920	0.84	NA	4528.15	0.285714
V19	19	tree	NA	NA	SP	0	0
V23	23	tree	NA	NA	SP	0	0
V25	25	tree	NA	NA	larch	0	0
V26	26	nest	8155	0	NA	0	0
V27	27	nest	160	0	NA	0	0
V28	28	tree	NA	0	NA	0	0

	node.id	type	sizes	canopy	treespecie	totalforag	norm.between
V1	1	nest	803	41.809	NA	93.5	0
V2	2	nest	3072	35.922	NA	217	0.27907
V3	3	nest	196	4.514	NA	0	0.27907
V4	4	nest	2284	38.66	NA	0	0.796512
V5	5	nest	188	0.636	NA	0	0.145349
V6	6	nest	5512	21.331	NA	185.85	0.744186
V7	7	nest	4650	21.331	NA	0	0.447674
V8	8	nest	11854	21.264	NA	200.8	0.145349
V9	9	nest	3854	0.976	NA	0	0
V10	10	nest	13275	40.079	NA	1300	1
V11	11	nest	14044	40.079	NA	0	0.145349
V12	12	nest	47810	0	NA	0	0
V13	13	nest	7745	29.807	NA	650.65	0.901163
V14	14	nest	19599	17.092	NA	205	0.889535
V15	15	nest	5035	43.958	NA	0	0.145349
V16	16	nest	7201	61.375	NA	529.39	0.011628
V17	17	nest	2284	51.555	NA	0	0
V18	18	nest	16492	8.446	NA	527.05	0.72093
V19	19	nest	130860	42.166	NA	2815.67	0.418605
V23	23	nest	19940	44.803	NA	0	0
V25	25	tree	NA	NA	SP	0	0
V26	26	tree	NA	NA	SP	0	0
V27	27	tree	NA	NA	SP	0	0
V28	28	tree	NA	NA	SP	0	0
V29	29	tree	NA	NA	SP	0	0
V30	30	tree	NA	NA	SP	0	0
V31	31	tree	NA	NA	SP	0	0

	node.id	type	sizes	canopy	treespecie	totalforag	norm.between
V2	2	nest	2641	23.05	NA	180.11	0.611511
V5	5	nest	388	20.02	NA	0	0
V6	6	nest	7095	30.05	NA	261.45	0.769784
V7	7	nest	315	27.17	NA	0	0
V8	8	nest	4927	32.49	NA	200.8	0.093525
V9	9	nest	1119	18.89	NA	0	0.856115
V10	10	nest	11761	22.15	NA	577.2	1
V11	11	nest	5768	24.87	NA	0	0.690647
V12	12	nest	12039	0.75	NA	0	0.611511
V13	13	nest	4810	37.25	NA	1137.5	0.151079
V18	18	nest	38014	38.71	NA	184.15	0.151079
V19	19	nest	949	26.02	NA	1351.25	0.553957
V20	20	nest	39929	43.4	NA	1057.65	0.42446
V21	21	nest	1778	16.81	NA	0	0
V23	23	nest	5539	41.3	NA	0	0.021583
V26	26	tree	NA	NA	SP	0	0
V27	27	tree	NA	NA	SP	0	0
V28	28	tree	NA	NA	SP	0	0
V29	29	tree	NA	NA	SP	0	0
V30	30	tree	NA	NA	SP	0	0
V31	31	tree	NA	NA	SP	0	0
V32	32	tree	NA	NA	SP	0	0
V33	33	tree	NA	NA	SP	0	0

	node.id	type	sizes	canopy	treespecie	totalforag	norm.between
V2	2	nest	145169	NA	NA	145.39	0.285714
V6	6	nest	13903	38.16	NA	157.5	1
V7	7	nest	4099	5.14	NA	0	0
V9	9	nest	10321	22.32	NA	0	0.714286
V10	10	nest	43942	28.34	NA	743.6	0.714286
V11	11	nest	3423	29.79	NA	0	0
V12	12	nest	13134	0.73	NA	0	0
V19	19	nest	20511	3.26	NA	1732.15	0.857143
V20	20	nest	166399	46.31	NA	899.1	0.285714
V22	22	nest	132504	58.76	NA	0	0
V26	26	tree	NA	NA	SP	0	0
V27	27	tree	NA	NA	SP	0	0
V28	28	tree	NA	NA	SP	0	0
V30	30	tree	NA	NA	SP	0	0
V31	31	tree	NA	NA	SP	0	0
V32	32	tree	NA	NA	SP	0	0

	node.id	type	sizes	canopy	treespecie	totalforag	norm.between
V2	2	nest	1938	31.73	NA	145.39	1
V9	9	nest	484	11.39	NA	0	0
V10	10	nest	1699	35.28	NA	171.6	0.642857
V11	11	nest	395	13.41	NA	649.89	0.285714
V19	19	nest	21714	45.31	NA	378.9	0.214286
V20	20	nest	18334	40.92	NA	1350	0.285714
V26	26	tree	NA	NA	SP	0	0
V27	27	tree	NA	NA	SP	0	0
V30	30	tree	NA	NA	SP	0	0
V31	31	tree	NA	NA	SP	0	0
V32	32	tree	NA	NA	SP	0	0
V34	34	nest	1628	13.23	NA	0	0
V35	35	nest	2468	42.98	NA	222.5	0.285714
V36	36	nest	1045	27.3	NA	0	0
V37	37	nest	245	44.31	NA	0	0

	node.id	type	sizes	canopy	treespecie	totalforag	norm.between
V2	2	nest	14664	36.29	NA	542.5	0.677419
V9	9	nest	119	0	NA	0	0.258065
V10	10	nest	1995	33.52	NA	431.6	0.806452
V11	11	nest	3649	20.01	NA	849.56	1
V19	19	nest	56276	42.72	NA	3817.65	0.677419
V20	20	nest	27509	45.98	NA	899.1	0
V26	26	tree	NA	NA	SP	0	0
V27	27	tree	NA	NA	SP	0	0
V28	28	tree	NA	NA	SP	0	0
V30	30	tree	NA	NA	SP	0	0
V31	31	tree	NA	NA	SP	0	0
V32	32	tree	NA	NA	SP	0	0
V34	34	nest	1858	13.64	NA	267.96	0.258065
V35	35	nest	8945	1.75	NA	119.26	0.367742
V36	36	nest	737	30.18	NA	0	0
V37	37	nest	793	43.15	NA	0	0
V38	38	nest	668	33.71	NA	0	0
V39	39	nest	7616	36.17	NA	206.36	0.677419
V40	40	nest	949	26.67	NA	0	0
V41	41	nest	256	21.12	NA	167	0
V42	42	nest	227	0.99	NA	0	0.135484
V43	43	nest	89	8.71	NA	0	0
V44	44	nest	754	30.43	NA	93.8	0.135484

	node.id	type	sizes	canopy	treespeci€	totalforag	norm.between
V1	1	nest	82098	27.533	NA	353	0.255814
V2	2	nest	87361	20.332	NA	529.2	0.255814
V3	3	nest	110959	21.904	NA	1300	1
V4	4	nest	145158	28.04	NA	425	0.837209
V5	5	nest	184308	34.684	NA	4370.45	0.488372
V6	6	nest	83326	35.692	NA	4001.5	0.488372
V8	8	tree	NA	NA	SP	0	0
V9	9	tree	NA	NA	oak	0	0
V10	10	tree	NA	NA	SP	0	0
V11	11	tree	NA	NA	oak	0	0
V12	12	tree	NA	NA	SP	0	0
V13	13	tree	NA	NA	SP	0	0
V14	14	tree	NA	NA	SP	0	0

	node.id	type	sizes	canopy	treespecie	totalforag	norm.between
V1	1	nest	2271	15.91	NA	882.5	0.516129
V2	2	nest	9902	16.43	NA	1402.8	0.290323
V3	3	nest	93535	31.84	NA	865.8	1
V4	4	nest	42719	37.27	NA	3183.05	0.741935
V5	5	nest	229374	43.29	NA	475	0.290323
V7	7	nest	2942	34.8	NA	775	0
V8	8	tree	NA	NA	SP	0	0
V9	9	tree	NA	NA	SP	0	0
V10	10	tree	NA	NA	SP	0	0
V12	12	tree	NA	NA	oak	0	0
V13	13	tree	NA	NA	SP	0	0

	node.id	type	sizes	canopy	treespecie	totalforag	norm.between
V3	3	nest	292205	38.99	NA	4950.9	0.555556
V4	4	nest	87252	32.73	NA	2875.45	1
V5	5	nest	230906	43.52	NA	3853.76	0.555556
V8	8	tree	NA	NA	SP	0	0
V9	9	tree	NA	NA	oak	0	0
V10	10	tree	NA	NA	SP	0	0
V11	11	tree	NA	NA	oak	0	0
V12	12	tree	NA	NA	SP	0	0
V13	13	tree	NA	NA	SP	0	0
V14	14	tree	NA	NA	SP	0	0

	node.id	type	sizes	canopy	treespecie	totalforag	norm.between
V3	3	nest	159647	37.78	NA	130	0.75
V4	4	nest	43246	40.93	NA	5253.5	1
V5	5	nest	240329	35.54	NA	4676.35	0.390625
V8	8	tree	NA	NA	SP	0	0
V9	9	tree	NA	NA	oak	0	0
V10	10	tree	NA	NA	SP	0	0
V11	11	tree	NA	NA	oak	0	0
V12	12	tree	NA	NA	SP	0	0
V13	13	tree	NA	NA	SP	0	0
V14	14	tree	NA	NA	SP	0	0
V15	15	nest	4446	35.09	NA	783.35	0.203125
V16	16	nest	11455	35.2	NA	4175	0
V17	17	nest	2271	23.51	NA	1749.5	0.828125
V18	18	nest	1805	22.93	NA	306.19	0.203125
V19	19	nest	3786	30.6	NA	170	0

	node.id	type	sizes	canopy	treespecie	totalforag	norm.between
V3	3	nest	51088	34.6	NA	9977.75	0.5
V4	4	nest	71040	27.47	NA	10031.4	1
V5	5	nest	51088	27.99	NA	5310	0.5
V8	8	tree	NA	NA	SP	0	0
V9	9	tree	NA	NA	oak	0	0
V10	10	tree	NA	NA	SP	0	0
V11	11	tree	NA	NA	oak	0	0
V12	12	tree	NA	NA	SP	0	0
V13	13	tree	NA	NA	SP	0	0
V14	14	tree	NA	NA	SP	0	0
